# Understanding the structural basis of substrate recognition by *Plasmodium falciparum* plasmepsin V to aid in the design of potent inhibitors

**DOI:** 10.1038/srep31420

**Published:** 2016-08-17

**Authors:** Rajiv K. Bedi, Chandan Patel, Vandana Mishra, Huogen Xiao, Rickey Y. Yada, Prasenjit Bhaumik

**Affiliations:** 1 Department of Biosciences and Bioengineering, Indian Institute of Technology Bombay, Powai, Mumbai, 400076, India; 2 Department of Chemistry, Indian Institute of Technology Bombay, Powai, Mumbai, 400076, India; 3 Department of Molecular and Cellular Biology, University of Guelph, Guelph, Ontario, N1G 2W1, Canada; 4 Faculty of Land and Food Systems, University of British Columbia, 248-2357 Main Mall, Vancouver, BC V6T 1Z4, Canada

## Abstract

*Plasmodium falciparum* plasmepsin V (PfPMV) is an essential aspartic protease required for parasite survival, thus, considered as a potential drug target. This study reports the first detailed structural analysis and molecular dynamics simulation of PfPMV as an apoenzyme and its complexes with the substrate PEXEL as well as with the inhibitor saquinavir. The presence of pro-peptide in PfPMV may not structurally hinder the formation of a functionally competent catalytic active site. The structure of PfPMV-PEXEL complex shows that the unique positions of Glu179 and Gln222 are responsible for providing the specificity of PEXEL substrate with arginine at P3 position. The structural analysis also reveals that the S4 binding pocket in PfPMV is occupied by Ile94, Ala98, Phe370 and Tyr472, and therefore, does not allow binding of pepstatin, a potent inhibitor of most pepsin-like aspartic proteases. Among the screened inhibitors, the HIV-1 protease inhibitors and KNI compounds have higher binding affinities for PfPMV with saquinavir having the highest value. The presence of a flexible group at P2 and a bulky hydrophobic group at P3 position of the inhibitor is preferred in the PfPMV substrate binding pocket. Results from the present study will aid in the design of potent inhibitors of PMV.

Malaria is an infectious disease that is responsible for causing illness in an estimated 200 to 500 million people and results in an annual mortality of 1 to 2 million persons^1^. The disease is spread through the transmission of unicellular eukaryotic protozoans of the genus *Plasmodium* with five known species *P. falciparum*, *P. vivax*, *P. ovale*, *P. malariae* and *P. knowlesi*^2^. *P. falciparum* and *P. vivax* being the deadliest, causing nearly all of the malaria related deaths in Africa and outside Africa^3^,^4^. Despite the availability of several effective antimalarial drugs such as chloroquine, sulfadoxine and artemisinin, the recent increased drug resistance of the malaria parasite^5^ necessitates an urgent need for designing new antimalarial compounds aimed at novel targets.

The life cycle of the parasite is complex, and begins with its asexual growth phase in humans after its invasion into red blood cells (RBCs), where the species differentiates (ring stage), metabolizes hemoglobin (Hb) (trophozoite stage) then replicates (schizont stage) over the course of 48 hours and finally is released into the bloodstream as a result of the rupturing of the host cells^6^,^7^,^8^. The disease symptoms appear upon the replication of the parasite inside erythrocytes. During the trophozoite stage, 75% of Hb content is degraded in order to produce amino acids required for protein synthesis and metabolic pathways^9^,^10^,^11^. Within the erythrocyte, the parasite resides inside a parasitophorous vacuole (PV). The ingestion of hemoglobin is carried out through an invagination called cytostome spanning the plasma membrane of the parasite, and PV membrane. The cytostome then fuses with a digestive vacuole which is acidic (pH range between 5 and 5.4) to release the filled Hb, where it is degraded^12^,^13^,^14^. In addition, a number of parasite proteins are exported across its plasma membrane, the surrounding PV membrane, and into the erythrocyte, thus altering the properties of the host cell^15^,^16^. These exported proteins are essential for parasite survival^17^ and virulence^18^,^19^.

The different classes of proteases involved in digesting hemoglobin include a cysteine protease (falcipain)^20^, a metalloprotease (falcilysin)^21^ and a family of aspartic proteases, the plasmepsins (PMs)^22^,^23^. Plasmepsins play a key role in a wide variety of cellular processes from hemoglobin degradation to the export of *Plasmodium* proteins which are essential for parasite growth/survival. Ten plasmepsins (PMI, II, IV and histo-aspartic protease as well as PMV-X) have been identified in the genome of *P. falciparum.* The four highly homologous *P. falciparum* aspartic proteases, PMI, II, IV and histo-aspartic protease (HAP) have been shown to be involved in hemoglobin degradation and have been considered as potential targets for antimalarial drug development^22^,^24^,^25^,^26^. Among PMV-X, only PMV has been characterized recently, showing much less sequence identity to other plasmepsins while having a role in exporting malarial proteins to the host cell^27^,^28^,^29^.

Results have shown that *P. falciparum* PMV (PfPMV) resides in the endoplasmic reticulum and is an essential protein for the survival, development and virulence of the parasite in the erythrocyte^27^,^28^, thus making this enzyme a potential target for developing antimalarial drugs. Biochemical studies^27^,^30^ and sequence analysis^28^,^31^ have shown that PfPMV is an aspartic protease with several important amino acid substitutions in the substrate binding pocket in contrast to other proteins belonging to pepsin-like aspartic protease family. PfPMV is involved in cleaving the conserved motif called PEXEL (*Plasmodium* EXport ELement) of the exported proteins and the motif is reported to be conserved among the *Plasmodium* sp. The PEXEL motif consists of a conserved sequence RxLxE/Q/D where it has been observed that the presence of arginine and leucine are important for substrate recognition^27^. Despite the presence of an active site signature motif of a pepsin-like aspartic protease, initial studies using solubilised PfPMV from parasites indicated no inhibition by pepstatin^28^ although immunoprecipitated HA-tagged PfPMV showed partial inhibition by HIV-1 protease inhibitors saquinavir, lopinavir and ritonavir^27^. Recent studies on mature recombinant PfPMV (Asp84-Asn521) indicated a 50% inhibition of the enzyme by HIV-1 protease inhibitor nelfinavir and a 10% inhibition by pepstatin^32^. Our studies on the recombinant PfPMV have indicated that the enzyme is active as a mature and truncated zymogen (Glu37-Asn521). Enzymatic activity of the recombinant PfPMV is partially inhibited by pepstatin and several KNI series inhibitors, but Cu^2+^ and Hg^2+^ ions strongly inhibited the activity^30^. Therefore, structural studies on PfPMV are essential in order to understand both the active site architecture of this enzyme and the structural basis of its substrate specificity. Homology modeling studies of PMVs from *P. falciparum* and *P. vivax* have been done and some important features of the active site of this enzyme have been revealed^31^,^33^. During the preparation of this manuscript the crystal structure of plasmepsin V from *P. vivax* (PvPMV) has been determined as complexed with an inhibitor^34^. The enzyme from *P. vivax* is smaller compared to its counterpart from *P. falciparum* and their primary sequences have important differences^33^. The reported structure also has around 30 missing residues in the region (Arg241 to Glu272) with large differences between the two enzymes. Structural studies on PfPMV would aid in understanding the mechanism of action of this enzyme.

In the present study, a structural model of *P. falciparum* PMV (PfPMV) has been developed. The structural model of PfPMV was used to find possible potential inhibitors of this enzyme. The docking-based screening method identified saquinavir as the best inhibitor among the tested compounds. In addition, the PEXEL substrate with the sequence “RTLVD” was also docked in the PfPMV active site. Molecular dynamics (MD) simulations of apo-PfPMV as well as its complexes with saquinavir and a PEXEL substrate were conducted. The PfPMV model complexed with PEXEL substrate suggested the enzyme’s specificity towards the substrate with Arg, Leu and Glu/Asp/Gln at P3, P1 and P2′ positions, respectively. Further, a structural analysis pointed to key features of the PfPMV active site that might explain the functional properties of this enzyme; hence the results of the study will facilitate the design of antimalarial drugs that target PMV.

## Results and Discussion

### Overall structural fold of PfPMV and its sequence comparison

**Figure 1:**
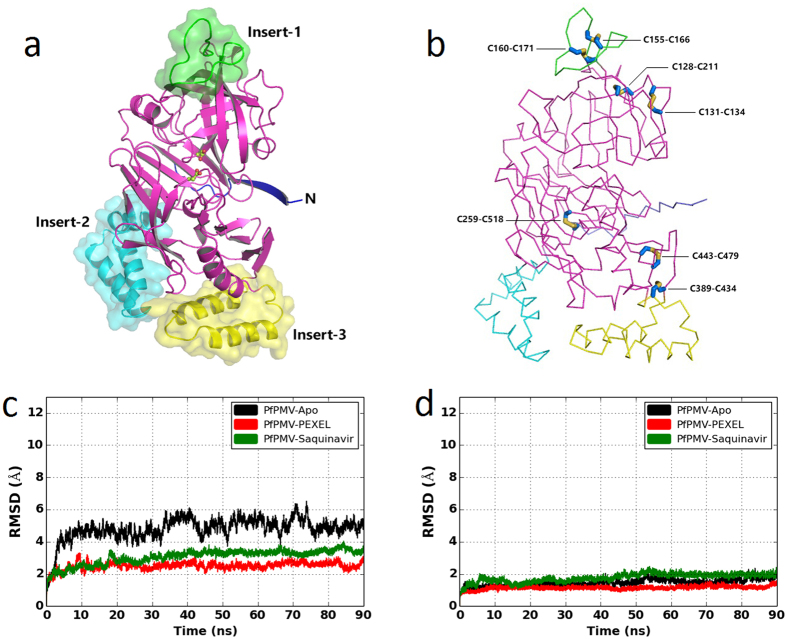
Overall structural fold of *P. falciparum* PMV (PfPMV). (**a**) The structure of truncated PfPMV zymogen is represented as cartoon. The polypeptide having classical pepsin-like aspartic protease fold is shown in magenta color. Three insertion domains, Insert-1, Insert-2 and Insert-3 are shown as green, cyan and yellow color with the semi-transparent surfaces, respectively. Part of the prosegment is shown in blue color. Two catalytic aspartates are shown as ball and stick model. (**b**) Ribbon structure of the truncated PfPMV zymogen with disulfide bonds are represented as sticks. Color coding of the polypeptide chain is same as panel a. (**c**) Plot of backbone r.m.s.d. change for overall structures of apo-PfPMV (black), PfPMV-PEXEL complex (red) and PfPMV-saquinavir complex (green) during the simulation. (**d**) Plot of backbone r.m.s.d. change only for the core structure (within 15.0 Å of the active site) of apo-PfPMV (black), PfPMV-PEXEL complex (red) and PfPMV-saquinavir complex (green) during the simulation process.

**Figure 2:**
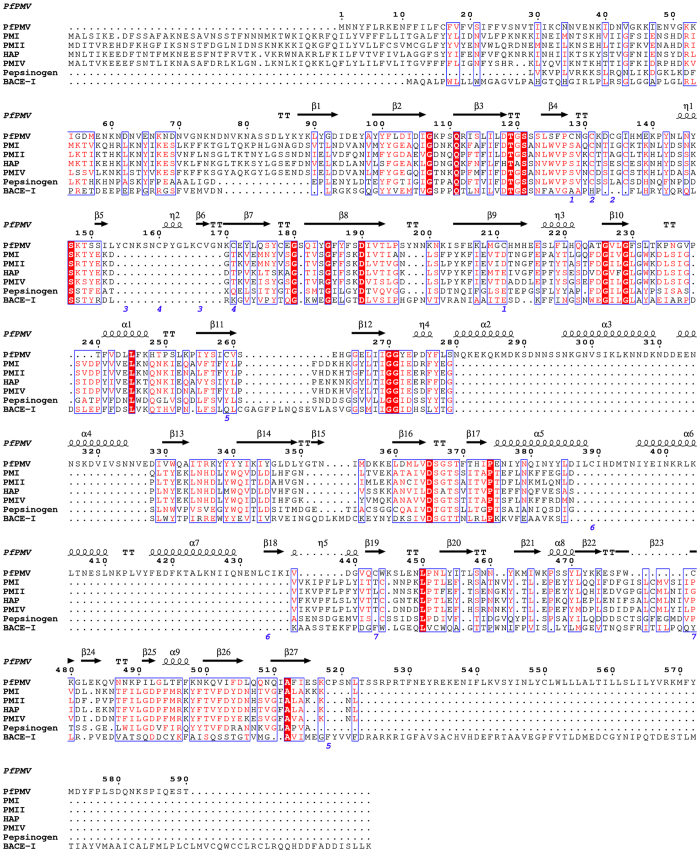
Sequence comparison of PfPMV with other pepsin-like aspartic proteases. Sequence alignment of PfPMV with vacuolar plasmepsins (PMI, PMII, PMIV and HAP), porcine pepsinogen and human β-secretase I (BACE-I). Secondary structural elements of PfPMV homology model is shown on top of the sequence alignment. The identical residues identified by ESPript (www.espript.ibcp.fr) are presented in the red background and the similar residues are shown as red color. The disulfide bonds present in PfPMV structure are shown by blue numbers at the bottom of the alignment.

**Figure 3:**
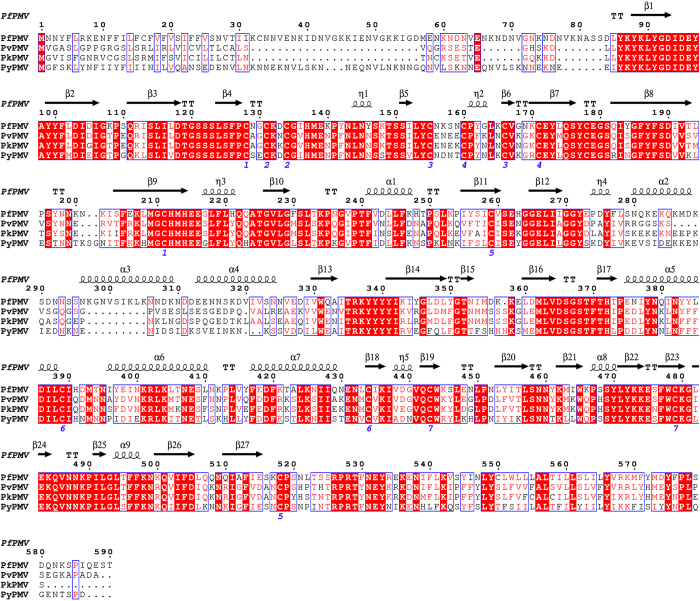
Sequence alignment of PMVs from human malaria causing parasites. The *Plasmodium* sp. in this alignment are *P. falciparum* (Pf), *P. vivax* (Pv), *P. knowlesi* (Pk) and *P. yoelii* (Py). The identical residues are shown in red background and the similar residues are shown as red color.

In the present study, the structural model of *P. falciparum* plasmepsin V (PfPMV) zymogen (Glu65-Leu522) was generated. The best-predicted structural model of truncated PfPMV zymogen presented in this study has C-score and TM-score of −0.41 and 0.66 ± 0.13, respectively. These values imply that the homology model of PfPMV have the right structural fold as the C-score of a predicted structure by I-TASSER^35^ typically lies in the range of −5 to 2 while a TM-score value greater than 0.5 is an indicator of the correctness of the predicted structural topology. The PfPMV homology model structure was compared with PMI (PDB ID = 3QRV), PMII (PDB ID = 1XDH), histo-aspartic protease (PDB ID = 3FNT), PMIV (PDB ID = 1LS5), human pepsin (PDB ID = 1PSO) and human β-secretase I (BACE-I) (PDB ID = 4GID) with the structural superposition producing root mean square deviation (r.m.s.d.) values of 2.1, 2.1, 2.0, 2.3, 2.0 and 2.0 Å, respectively. These structural superimposition results indicate that the overall predicted structural fold of PfPMV is similar to that of pepsin-like aspartic protease (Figs 1 and S1a) despite having a very low sequence identity with the proteins belonging to the family of pepsin-like aspartic protease^34^. However, PfPMV differs from the structures of other pepsin-like aspartic proteases in that it has three additional structural insertions. Sequence comparison (Fig. 2) reveals that PfPMV has two conserved active site regions (DTGS and DSGS) providing two catalytic aspartate residues (Asp118 and Asp365). The DTGS motif present at the N-terminal domain is highly conserved except in histo-aspartic protease^22^. The truncated PfPMV zymogen structure has two domains mainly composed of β-sheets and one small satellite domain (Insert-2). The wide substrate binding active site cleft is present in between the two domains. The prosegment is in the extended conformation and it may not interact with the active site residues (Fig. 1), hence, allowing PfPMV zymogen to remain in a catalytically active conformation. Recent reports^30^,^32^ on enzymatically active PfPMV zymogen support this structural model having a hinge region (Lys79-Ser83) which connects the prosegment with the mature enzyme. The hinge region is distant from the active site, therefore, may not be accessible for cleavage during auto-activation. This structural arrangement supports the reported observation that the PfPMV zymogen gets activated *via* proteolytic action mediated by other proteases and not by autolysis at acidic pH^30^. This structural model is also in agreement with the enzymatic activity of the GFP-tagged, full length PfPMV zymogen^29^ indicating no inhibition of its function by the propeptide, i.e., dissimilar to other pepsin-like aspartic protease inactive zymogens. However, the molecular dynamics simulation of PfPMV zymogen is essential in order to understand the interactions of propeptide with the mature enzyme. In this study, the mature enzyme is composed of residues Ser82-Leu522. Recent experimental data^30^ also identified that the cleavage of the prosegment to form mature protein occurs between Asn80 and Ala81. Another study indicated that enzymatically efficient, mature PfPMV is composed of residues Asp84-Asn521^32^. The mature PfPMV structural models as apo-, complexed with PEXEL substrate and saquinavir inhibitor complex have been simulated for 90 ns. The three final structures exhibited good stereochemical geometry of the residues as analyzed by Ramachandran maps (Fig. S2a–c). Approximately 94% residues of these structures are present in the most favoured regions of the Ramachandran map. The sampled average structures also show good stereochemical geometry as analyzed by Ramachandran map (Fig. S2d–f). Few outliers, which are present in the flexible loops of the structures, have also been identified. The structures also remained stable during the simulation process as revealed by the r.m.s.d. plots (Fig. 1c,d). The average PfPMV structures have been analyzed in order to assign secondary structural elements and domain arrangements of the mature enzyme. The N-terminal domain of mature PfPMV is composed of residues Ser82-Ala333 and Lys500-Leu522. This domain contains a small insertion (Insert-1) composed of 19 residues (Leu153-Cys171). A similar insertion was also proposed by Guruprasad *et al*.^31^ in the PfPMV structural model. The N-terminal domain also has a small satellite domain composed of residues Leu278-Val326 (Insert-2). This small domain is formed because of an insertion of a region (Insert-2) in the PfPMV polypeptide chain compared to other members of pepsin-like aspartic protease family (Figs 1 and 2). This small satellite domain is mainly composed of helices and has a helix-turn-helix structural fold (Figs 1a and S3a). Sequence comparison (Fig. 3) reflects the Insert-2 domain to be smaller in PMVs from other human malaria causing *Plasmodium* sp. The structure and sequence analysis also reveals that PfPMV Insert-2 region has nine lysine residues which contribute towards the positive charged surface of this domain. The secondary structural elements present in this domain are quite stable (Fig. S3c) during the entire simulation process. Molecular dynamics simulations of apo- as well as complexed PfPMV structures (Fig. S4) show that this domain is highly flexible in nature with respect to the core structure (Fig. S1c) and it may be a reason for lack of electron density of the Insert-2 region in PvPMV crystal structure (Fig. S1b) although both the enzymes have very similar structural fold^34^. The N-terminal domain of the PfPMV structure also has the “flap” region (Glu172-Phe186) which may play a crucial role for the substrate binding. The flap contains the conserved Tyr177 (except in HAP, Fig. 2) and the corresponding tyrosine residue has been proposed to play an important role in the catalysis of pepsin-like aspartic proteases^36^. Although most of the residues of the flap regions are conserved in vacuolar plasmepsins of human malaria parasites, PfPMV has some notable substitutions. One important substitution in PfPMV is Tyr173 at the position of Val/Leu of pepsin-like aspartic proteases. The flap of PfPMV also has one extra residue (Ser181) compared to other members of the same family. However, most of the residues in flap region are well conserved in PMVs of human malaria causing parasites. The C-terminal domain of PfPMV is composed of Tyr339-Leu494. This domain has another insertion (Insert-3) region composed of residues Ile387-Cys434. This insertion also has a helix-turn-helix fold with two α-helices. Presence of the similar secondary structural elements (Fig. S1b) have also been reported for the crystal structure of PvPMV^34^. The sequences of this region among PMVs are well conserved with a few differences and have similar lengths in the malaria causing parasites; therefore, it is very likely that the secondary structural elements of Insert-3 regions also remain conserved among all the enzymes. The simulation results suggest that this domain is not as flexible as the Insert-2 domain (Fig. S4). However, careful analysis shows that several lysine and arginine residues are present on the surface of this domain attributing towards the positively charged surface of this insertion region (Fig. S3b). The junction of two domains of PfPMV is assembled into a characteristic six-stranded interdomain β-sheet which serves to link the two domains together and this structural feature is conserved in the pepsin-like aspartic protease family^23^.

The PfPMV structure has a total of seven disulfide bridges as presented in Fig. 1b. The first two disulfide bonds (Cys128-Cys211 and Cys131-Cys134) are present in the N-terminal domain. The second pair of disulfide bridges (Cys155-Cys166 and Cys160-Cys171) are present in the Insert-1 domain. The remaining three (Cys259-Cys518, Cys389-Cys434 and Cys443-Cys479) are located in the C-terminal domain. The same number of disulfide linkages have been reported in the crystal structure of PvPMV^34^. The disulfide bond-forming cysteines are conserved in the PMVs from malaria-causing parasites (Fig. 3), hence these bonds are expected to be conserved in all PMV structures. Our simulation data (Fig. S5) show that the distance between the Cα atoms of the cysteine residues involved in formation of the disulfide bonds are stable during the simulation in the three structures. These distances are also comparable to those observed in the crystal structure of PvPMV. This result may indicate that the disulfide bonds are important to maintain the structural integrity of the PMVs from different *Plasmodium* species. It is also very important to note that although PfPMV has a high degree of structural similarity with pepsin-like aspartic proteases, the vacuolar plasmepsins and mammalian pepsins have only two and three disulfide bonds, respectively, in their structures.

Two insertion domains (Insert 2 and 3) of PfPMV have α-helical structural folds and these structural features may be comparable to the plant specific insertion saposin-like domains present in plant aspartic proteases^37^. The saposin-like domain of potato aspartic protease has a helix-turn-helix motif and has been proven to interact with membrane^38^. Unlike other vacuolar plasmepsins, the PfPMV resides in the endoplasmic reticulum of the parasite and remains anchored by its putative C-terminal domain^28^. Based on the structural architecture and charge distribution of Insert-2 and 3 regions of PfPMV, it is likely that these two domains are involved in interaction with the endoplasmic reticulum membrane and may have originated from the plant aspartic proteases.

### Architecture of the apo-PfPMV active site

**Figure 4:**
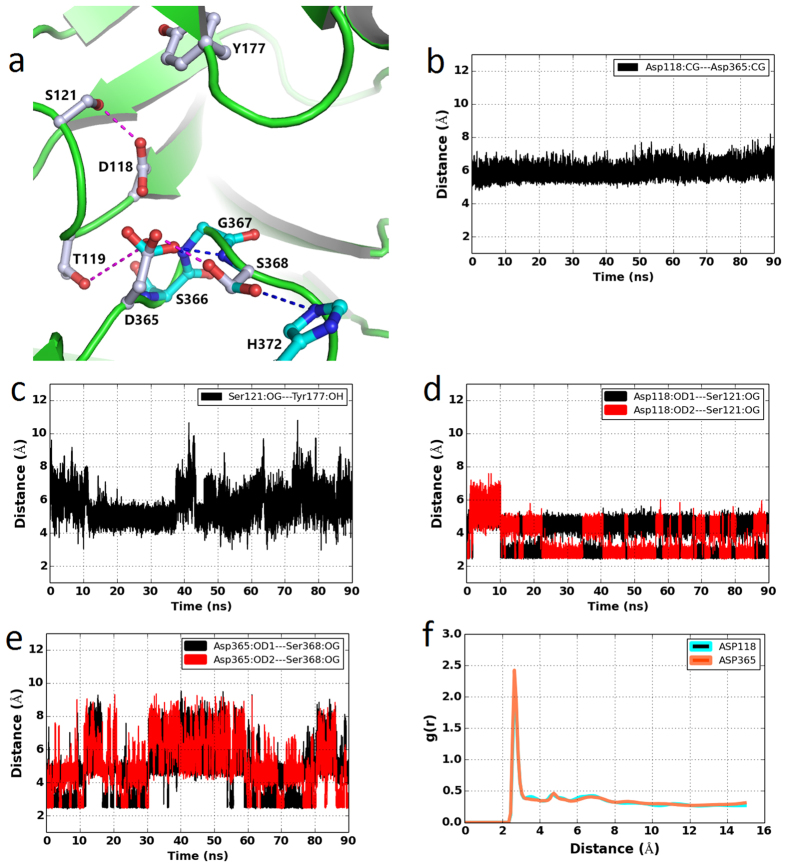
Important interactions present in the apo-PfPMV active site. (**a**) Zoomed in view of apo-PfPMV active site. Putative catalytic residues are shown as ball and stick model with grey color carbon and hydrogen bonding interactions are shown as purple dotted lines. The alternative conformations of Asp365, Ser368 and their interacting partners are shown with cyan color carbon ball and stick model; and corresponding hydrogen bonds are presented as blue lines. (**b**) The distance between CG atoms of two catalytic aspartates (Asp118 and Asp365) during simulation. (**c**) The distance between the Tyr177 hydroxyl group oxygen (OH) and Ser121 hydroxyl group oxygen (OG) during simulation. (**d**) The distance between the carboxylate oxygen atoms (OD1 and OD2) of Asp118 and Ser121 hydroxyl group oxygen atom (OG) during simulation. (**e**) The distance between the carboxylate oxygen atoms (OD1 and OD2) of Asp365 and Ser368 hydroxyl group oxygen atom (OG) during simulation. (**f** ) The radial distribution function [g(r)] of water hydrogen atoms around the carboxylate oxygen atoms of Asp118 and Asp365 during simulation.

Structural model and sequence comparison shows that PfPMV belongs to the family of pepsin-like aspartic proteases. The apo-PfPMV structure was simulated for 90 ns and the final structure exhibited good stereochemical geometry of the residues as analyzed by Ramachandran map (Fig. S2a). The stability of the apo-structure was also monitored by r.m.s.d. change during the entire simulation (Fig. 1c,d). The stability of the system has also been monitored during the entire simulation process (Fig. S6) by the change in energies of the systems. A snapshot structure of the apo-PfPMV with the lowest r.m.s.d. value, with respect to the average structure from the last 20 ns of the simulation trajectory, was used to analyze the critical interactions in the active site. The active site architecture of the apo-enzyme remained quite stable during the simulation (Figs 4 and S4a) without major changes among the important residues involved in catalysis. This was also reflected in the lower r.m.s.d. values of the core structure compared to the overall structure during the simulation. The Cγ atoms of the carboxylate groups of Asp365 and Asp118 are at a distance of around 5.8 Å (Fig. 4b) which is comparable to the corresponding distance reported for the crystal structure of pepsin-like aspartic proteases^39^ and PvPMV^34^. Movements occurred in the flap region (Fig. 4c) during the simulation process. Movement of the flap in the unliganded vacuolar plasmepsin structures has also been reported recently by molecular dynamics simulation^40^. Molecular dynamics simulation of apo-β-secretase also revealed significant movement of the flap and other loop regions during the simulation^41^. The analysis of domain movement (Fig. S4a) of the apo-PfPMV structure revealed that three insertion domains moved during the simulation and the core of the structure remained stable. Two catalytic aspartates (Asp118 and Asp365) are at a favourable distance to participate in catalysis and other interactions support correct ionization states of the side chain carboxylate groups of these residues (Fig. 4a,d,e). The carboxylic acid group of Asp365 side chain predominantly forms hydrogen bond with the side chain −OH group of Ser368 (Fig. 4e). This hydrogen bond is maintained for almost 52% of the time of the simulation process as Ser368 side chain also forms hydrogen bond with the side chain of His372. In the crystal structure of PvPMV-inhibitor complex (PDB ID = 4ZL4), the equivalent serine residue (Ser316) is hydrogen bonded to the side chain of His320. The carboxylate group of Asp365 also forms hydrogen bonds with the main chain –NH groups of Gly367 (30% time) and Ser368 (12% time). Similar stability of the hydrogen bond formed between Asp214 and Thr217 has been reported previously in apo-PMII structure simulation^42^. The residue corresponding to PfPMV Ser368 (Fig. 2) in pepsin-like aspartic proteases and human BACE-I is a conserved threonine (Thr218, pepsin numbering); and this threonine has been proposed to play a crucial role in the catalysis^36^. The presence of water molecules around Asp118 and Asp365 were monitored during the simulation (Fig. 4f) and the results showed that water molecules were present around the active site aspartates. The water molecule close to Asp365 may act as the nucleophile for the peptide bond cleavage similar to that proposed as the catalytic mechanism for the other pepsin-like aspartic proteases. On the other side of the active site of PfPMV, Asp118 is hydrogen bonded to Ser121 (Fig. 4d). This hydrogen bonding interaction is maintained for almost 90% of the time of the entire simulation period. This serine residue is conserved in the pepsin-like aspartic protease family. In pepsin, this serine (Ser37) also takes part in the proton relay mediated by Trp39, Tyr77 and a water molecule to increase the acidity of Asp32 side chain carboxylic acid group. In PfPMV, the position of Trp39 is occupied by Ser125. However, Tyr177 is present in PfPMV at the position of Tyr77. Closing of the flap may bring Tyr177 closer to the active site, thereby making it possible to contribute in a catalytic mechanism. Possible involvement of Tyr177 in substrate binding and catalytic function of PfPMV is discussed later. The opening and closing of the flap region (Figs 4c and S4a) is an indication that this secondary structural element of PfPMV structure is flexible and might be involved in substrate binding. In the pepsin-like aspartic protease family, closure of the flap due to substrate or ligand binding has been reported^23^,^43^. Opening and closing of the flap has also been reported in apo-PMI^23^.

### Mode of binding of PEXEL substrate in the PfPMV active site

**Figure 5:**
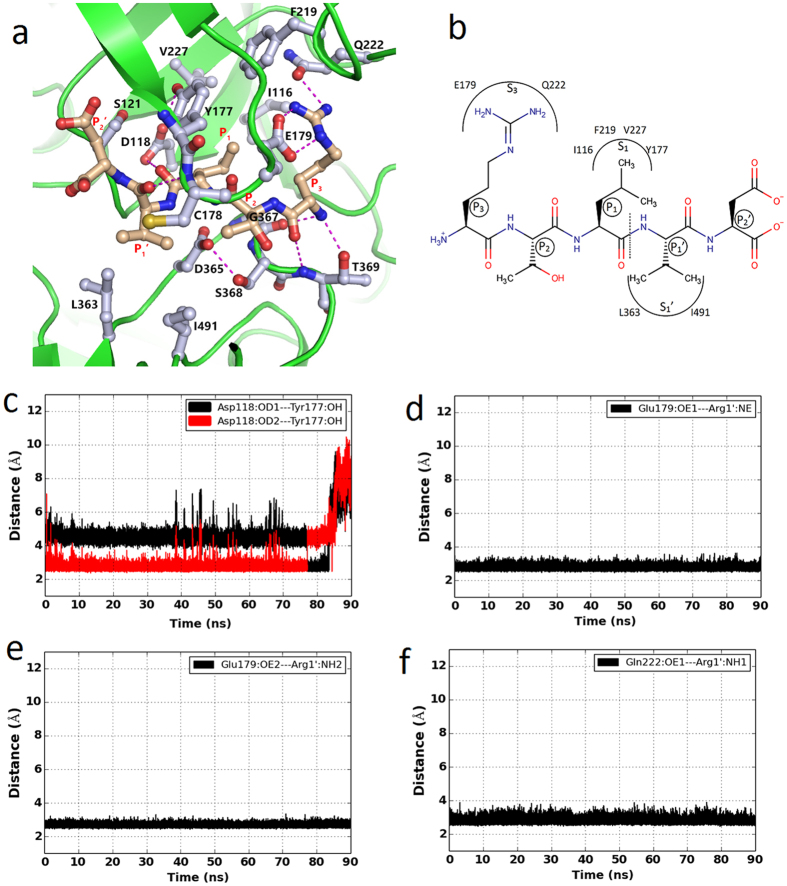
Binding mode of PEXEL substrate in the PfPMV active site. (**a**) Zoomed in view of substrate binding pocket of PfPMV. Bound PEXEL substrate (RTLVD) is shown as ball and stick with carbon colored as light brown. Residues involved in the substrate binding are shown as ball and stick with carbon colored as grey, and the hydrogen bonding interactions are presented as dotted lines. (**b**) Schematic representation of the PEXEL substrate binding pocket in PfPMV. The peptide cleavage site is indicated by a vertical dotted line. (**c**) The distance between the Tyr177 hydroxyl group oxygen atom (OH) and the carboxylate oxygen atoms (OD1 and OD2) of Asp118 during simulation. (**d**) The distance between the PfPMV Glu179 carboxylate oxygen atom (OE1) and PEXEL arginine (P3) side chain guanidium group nitrogen atom (NE) during simulation. (**e**) The distance between the PfPMV Glu179 carboxylate oxygen atom (OE2) and PEXEL arginine (P3) side chain guanidium group nitrogen atom (NH2) during simulation. (**f** ) The distance between the PfPMV Gln222 amide oxygen atom (OE1) and PEXEL arginine (P3) side chain guanidium group nitrogen atom (NH2) during simulation.

**Figure 6:**
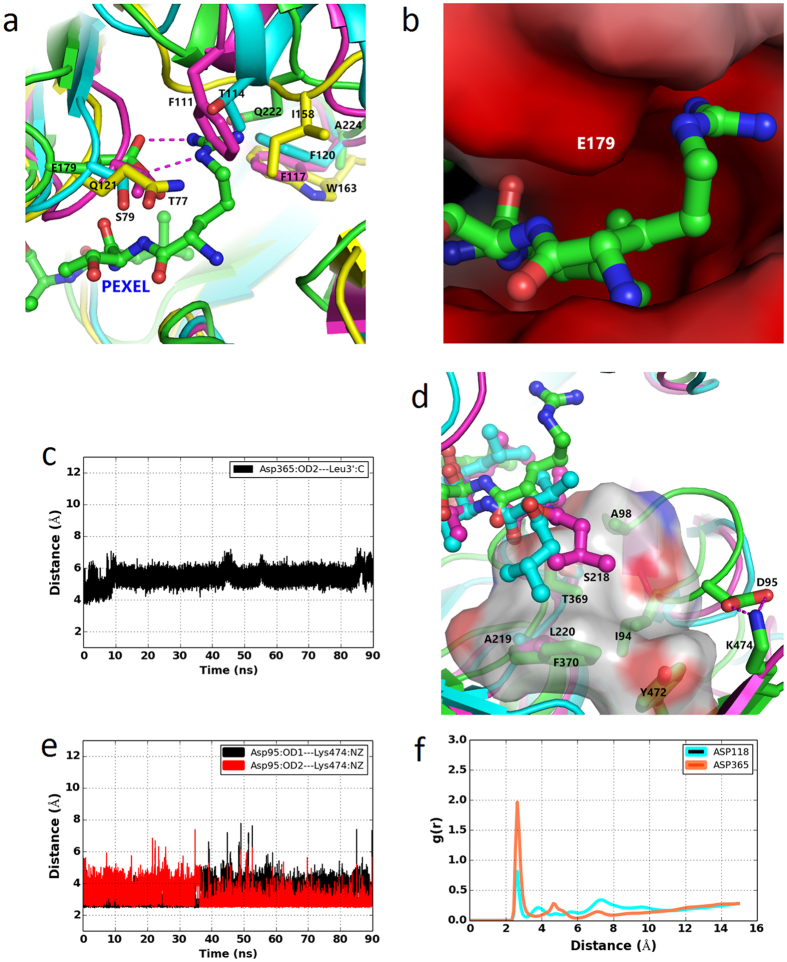
Structural basis of PEXEL substrate recognition by PfPMV. (**a**) Structural comparison of PfPMV-PEXEL complex (green) with PMII-pepstatin complex (cyan), pepsin-pepstatin complex (magenta) and ligand bound BACE-I (yellow) showing the differences in S3 binding pockets. The PEXEL substrate is shown as ball and stick model. Residues of proteins are shown as stick only. (**b**) Electrostatic potential surface of PfPMV showing the zoomed in view of the S3 substrate binding pocket. Negatively charged surface is shown in red color. Bound PEXEL substrate is shown as ball and stick. (**c**) The change of distance between the PEXEL leucine main chain carbonyl carbon (C) and carboxylate oxygen atom (OD2) of PfPMV Asp365. (**d**) Comparison of substrate binding pockets (S4) of PfPMV (green carbon) with that of PMII (cyan carbon) and pepsin (magenta carbon). The PEXEL substrate in PfPMV, pepstatin in both PMII and pepsin are shown as ball and stick. Important residues are shown as stick representation. Polar interactions are shown as dotted lines. The semi-transparent surface of the PfPMV S4 binding pocket is also shown. (**e**) The distance between the carboxylate oxygen atoms (OD1 and OD2) of Asp95, and Lys474 side chain amino group nitrogen atom (NΖ) during simulation. (**f** ) The radial distribution function [g(r)] of water hydrogen atoms around the carboxylate oxygen atoms of Asp118 and Asp365 during simulation.

In order to understand the interaction of PEXEL substrate with PfPMV, a molecular dynamics simulation of PfPMV-PEXEL complex was conducted. The PEXEL substrate was designed as polypeptide with sequence RTLVD. This peptide was docked in the mature PfPMV active site (affinity: −8.1 kcal/mol). Molecular dynamics simulation of the PfPMV-RTLVD complex shows the possible interactions of the bound substrate in the active site cleft of the enzyme. The stability of the PfPMV-PEXEL complex structure was monitored by the stereochemical geometry of the residues, change in energy of the system and r.m.s.d. change during the entire simulation (Figs S2, 1c,d and S9). A snapshot structure of the PfPMV-PEXEL complex with the lowest r.m.s.d. value, with respect to the average structure from the last 20 ns of the simulation trajectory, was used to analyze the important interactions in the active site and structural comparison with the other pepsin-like aspartic proteases. Figure 1c shows that fewer overall structural changes occur in the PfPMV-PEXEL complex structure compared to the apo-PfPMV structure. During the simulation process, movement is observed only in the Insert-2 region and other parts of the substrate bound structure are quite stable. The core of the complexed structure remains stable (Figs 1d and S4b) and accommodates the substrate in the active site. Unlike the apo-PfPMV, the flap in the substrate bound structure is in the closed conformation and the side chain of Tyr177 from the flap forms a stable hydrogen bond with the carboxylate group of Asp118 (Fig. 5a,c). This observation is in agreement with the fact that the flap in pepsin-like aspartic proteases adopts a closed conformation in substrate/ligand bound enzyme^44^. The mode of binding of the PEXEL substrate in the PfPMV active site (Figs 5a,b, S7a and S8) is similar to the observed mode of binding of pepstatin, a peptidomimetic inhibitor in vacuolar plasmepsins^23^,^43^. Arginine, the N-terminal residue (P3) of the PEXEL substrate is bound in a pocket (S3) formed primarily by side chains of Glu179 and Gln222. The main chain –NH and carbonyl groups of arginine are hydrogen bonded to the side chain hydroxyl and main chain –NH groups of Thr369, respectively (Fig. S7b,c). Towards the end of the simulation the carbonyl group of PEXEL Arg1 moves away from Thr369. The guanidine group of arginine forms a salt bridge interaction with side chain carboxylate group of Glu179 (Fig. 5d,e). The amide group of Gln222 forms a hydrogen bond with the substrate arginine (Fig. 5f) side chain. Structural comparisons of PfPMV with BACE-I, pepsin and vacuolar plasmepsin (PMII) indicate that despite having two anti-parallel β-strands at the beginning of these structures, the loop connecting the strands in PfPMV is longer and has two acidic residues (Asp95, Glu96) which partially contributes to the formation of the S3 pocket. The charge distribution shows that the PfPMV pocket that binds arginine (P3) of the PEXEL substrate is predominantly negatively charged (Fig. 6b), whereas the same pocket in PMII is more positively charged. The simulation results show that the presence of Glu179 at the tip of the flap is the key residue for providing specificity towards the arginine at the P3 position of the PEXEL substrate. Glu179 and Gln222 are conserved in PMVs of human malaria causing parasites (Fig. 3). Thus, identical interactions are likely to be present for substrate binding in other malaria causing parasite PMVs. Recently, the crystal structure of PvPMV has been determined complexed with an inhibitor having modified arginine at P3 position. The PvPMV-inhibitor complex also shows that Glu179 and Gln222 may help in binding PEXEL substrate in the enzyme active site^34^. To understand the structural basis of substrate specificity of PfPMV, the structure of PfPMV-PEXEL complex was superimposed with inhibitor bound complexes of pepsin, BACE-I and PMII. The position of PfPMV Gln222 is occupied by Thr114, Phe111 and Ile158 in PMII, pepsin and BACE-I, respectively (Fig. 6a.). Glu179 of PfPMV structure superposes with Ser79, Thr77 and Gln121 in PMII, pepsin and BACE-I, respectively. The upper part of the S3 substrate binding pocket in BACE-I, pepsin and PMII is filled with Trp163, Phe117 and Phe120, respectively. The same position is occupied by Ala224 in PfPMV which creates space for the positioning of the guanidine group of arginine. Because of these substitutions, pepsin, vacuolar plasmepsins and BACE-I do not act on the PEXEL substrate. The threonine at P2 position of the PEXEL substrate is pointing away from the core of the active site. However, the main chain –NH group of threonine forms hydrogen bonds with the carboxylate group of Glu179 (Fig. S7d). The S2 pocket is mainly formed by side chain of Cys178. Side chains of Cys178 and Glu179 (only the Cα and Cβ atoms) hydrophobically interact with the side chain methyl group of threonine at P2. The formation of an S2 pocket by these two residues has been predicted previously^45^. The S1 pocket accommodating leucine at the P1 position of PEXEL substrate is predominantly hydrophobic. The side chain of leucine is anchored inside the S1 pocket through hydrophobic interactions with side chains of Ile116, Tyr177, Phe219 and Val227 (Fig. 5a,b). These residues are conserved in PMVs of other malaria causing parasites. Hence, it may be proposed that these residues provide structural features for binding of the PEXEL substrate leucine which is strictly conserved at the P1 position. Some of the residues forming the S1 pocket had been previously proposed^31^,^45^ based on modeling studies. However, our simulation data show that a few previously predicted residues (Leu218, Ser368)^31^ do not interact with the substrate leucine. Another residue, Ile113, reported previously^45^ to be involved in formation of S1 pocket is not at the closer proximity of the substrate binding pocket. Analysis of the corresponding pocket in other vacuolar plasmepsins and pepsin shows that S1 pocket is also hydrophobic in those enzymes. Tyr177 is well conserved, except in HAP. Ile116 and Phe219 are replaced by similar amino acids. It is important to note that Val227 in PfPMV is replaced by Ile in other members except in HAP. The simulation results indicate that the carbonyl group carbon atom of the PEXEL substrate leucine is at a constant distance from the carboxylate group of Asp365 (Fig. 6c). The side chain of Asp365 is positioned properly by hydrogen bonding interaction (Fig. S7e) between its carboxylate group and side chain hydroxyl group of Thr119 which rotates away at the end of the simulation. Similar hydrogen bonding interaction between the side chains of Asp218 and Thr33 is also observed in the high resolution (1.4 Å) crystal structure (PDB ID = 3Q70) of a secreted aspartic protease from *Candida albicans* complexed with ritonavir. This spatial arrangement in the PfPMV active site favours placement of a water molecule between Asp365 carboxylate group and PEXEL leucine carbonyl group. The carbonyl group of the scissile bond points towards the Asp118. The distribution of water molecules reveals that Asp365 is more hydrated compared to Asp118 (Fig. 6f). The P1′ position of the substrate has valine and it has hydrophobic interactions with the side chains of Leu363 and Ile491. The side chain –SH group of Cys178 is in close proximity to the valine side chain. Hence, a small polar amino acid like serine is preferred in the natural PEXEL substrate. However, it has also been reported that PfPMV can cleave PEXEL substrate with valine at P1′ position^32^. The PEXEL substrate used in this study has an aspartate at the P2′ position. This position in the natural PEXEL substrate sequences is occupied by Glu, Asp or Gln. Based on the analyzed PEXEL sequences it has been reported that the probability of occurrence of Glu is highest and Gln is lowest at this position^46^. The S2′ pocket in PfPMV is formed by the side chains of Gln175, Lys234 and Tyr339 and thus provides the polar environment for binding of the side chains of Glu/Asp/Gln at P2′ position of the substrate. Gln175, Lys234 and Tyr339 are also conserved in PfPMVs from malaria-causing parasites.

The structure of PfPMV-PEXEL complex was superimposed with PMII-pepstatin complex (PDB ID = 1XDH) and pepsin-pepstatin complex (PDB ID = 1PSO) (Fig. S8). As seen in the pepstatin-complexed PMII and pepsin structures, the flap also adopts a closed conformation when the PEXEL substrate binds to the PfPMV active site. Despite having a pepsin-like aspartic protease active site signature motif, PfPMV is not inhibited by pepstatin^28^,^32^. The structural analysis (Figs S8 and 6d) reveals that PfPMV has three substitutions which may be responsible for preventing pepstatin from binding to its active site. The isovaleryl group at P4 position of pepstatin is positioned in such a way that it collides with the side chain of Phe370 in PfPMV. The corresponding residue in pepsin is leucine and in the vacuolar plasmepsins is Ser/Ala/Val/Thr. The S4 pocket in PfPMV is also filled by Tyr472. The position of Tyr472 is occupied by Leu276 in pepsin and Gln276 in PMII, thus providing space for the binding of pepstatin. Another important residue blocking the binding of the isovaleryl group is Ile94 which is present in the loop connecting the first two strands of mature PfPMV. Due to the presence of Asp95 and Glu96, this loop has a different conformation and the hydrophobic residues (Ile94 and Ala98) present in this loop blocks the S4 binding pocket. The different conformation of this loop is facilitated by a salt bridge between the side chains of Asp95 and Lys474 present in the C-terminal loop (Tyr472–Lys480) (Fig. 6d,e). This C-terminal loop is highly flexible and adopts a different conformation upon substrate binding in proteins belonging to pepsin-like aspartic proteases^43^. Ile94, Phe370 and Tyr472 are also conserved among PMVs of human malaria-causing *Plasmodium* sp. (Fig. 3). Because of the bulky hydrophobic side chain substitutions, the S4 pocket of PfPMV does not allow the binding of pepstatin. However, a PEXEL substrate with smaller side chain containing amino acid at S4 position will bind to PfPMV. The PfPMV-PEXEL complex structure was also compared with inhibitor bound BACE-I structures (PDB IDs = 4GID, 3LNK). Structural comparison reveals that the rigid phenyl group at the P2 position of the inhibitor is in close contact with Glu179. Due to the presence of a rigid phenyl group in the inhibitor, the rotation is restricted and thus may prevent tight binding of these inhibitors to the PfPMV active site.

Analysis of the PfPMV-PEXEL complex structure reveals that the distance between Asp365 carboxylate oxygen atom (OD2) and the carbonyl carbon atom (C) of PEXEL substrate is around 6.0 Å (Fig. 6c). This distance favours the placement of a water molecule between these two atoms for generation of hydroxyl anion as a nucleophile by Asp365 to initiate the first step of peptide hydrolysis reaction. Presence of a water molecule in the catalytically competent active site of the PfPMV-PEXEL complex active site is also observed (Fig. 6f). Water molecules are more populated around Asp365 than Asp118. The high resolution structure of penicillopepsin (PDB ID = 3APP)^47^ showed the presence of a water molecule between two catalytic aspartates (Asp32 and Asp215). It has been suggested that this water molecule starts the nucleophilic attack to the carbonyl carbon of the peptide bond during peptide hydrolysis^36^. As has been proposed previously for pepsin-like aspartic proteases, the presence of more water molecules around Asp365 is an indication that, Asp365 in PfPMV acts as a base to abstract the proton from the neighbouring water molecule and the resulting hydroxyl anion then starts the peptide hydrolysis reaction. During the simulation of the apo-PfPMV structure, the flap is flexible and adopts an open conformation. In the core of the active site of the PfPMV-PEXEL structure, the flap is in the closed conformation as a result of the PEXEL substrate binding (Fig. 5c), and the Tyr177 side chain hydroxyl group forms a hydrogen bond with the carboxylate group of Asp118. Formation of a direct hydrogen bond between the flap tyrosine and the active site aspartate has not been observed in any protein belonging to the family of pepsin-like aspartic proteases. This may have resulted from the replacement of conserved tryptophan (Trp39 in pepsin) by Ser125 in PfPMV. These hydrogen bonding networks may be responsible for maintaining reactivity of the two catalytic aspartates in the PfPMV active site. However, further QM/MM calculation would provide a detailed understanding about the exact proton transfer mechanism during the catalysis by PfPMV.

### Docking of inhibitors in the PfPMV active site

Several inhibitors of aspartic proteases were docked in the PfPMV active site. The binding affinity based on docking is presented in Table 1. The PEXEL substrate (RTLVD) was also docked (affinity: −8.1 kcal/mol) in the active site of PfPMV. Covalent structures of the docked inhibitors are presented in supplementary Table 1. Among the docked inhibitors, saquinavir has the highest affinity (−12.8 kcal/mol). Inhibition of PEXEL processing activity by PfPMV has been studied previously^27^ using lopinavir, nelfinovir, ritonavir and saquinavir. Among these HIV-1 protease inhibitors, saquinavir was the most effective in inhibiting PfPMV activity. Docking results also suggest that saquinavir has a better affinity compared to lopinavir, nelfinovir, and ritonavir. The comparison of binding affinity also reflects that these retroviral protease inhibitors have much higher affinity compared to pepstatin, and thus could be effective PfPMV inhibitors. Poor or no inhibition of native^28^ and recombinant^32^ PfPMV by pepstatin has also been reported. It is also important to note that a few tested KNI compounds have comparable affinities to saquinavir. The affinities of these KNI compounds are higher than potent BACE-I inhibitors (SCH743813, PB8, and BJC060). The KNI compounds have also been reported to inhibit recombinant PfPMV^30^. Low affinity of BACE-I inhibitors is also comparable to the experimental results showing almost negligible inhibition of PfPMV by these compounds^29^. The KNI inhibitors^48^ have also been proven to block the enzymatic activity of vacuolar plasmepsins^23^,^49^. Our docking results indicates that HIV-1 protease inhibitors and KNI compounds block the active site of PfPMV, hence, these inhibitors could be further modified to develop improved antimalarial drugs. Compounds having this scaffold could be tested biochemically for their inhibitory effect on PfPMV enzyme activity.

### Binding mode of saquinavir in the PfPMV active site

**Figure 7:**
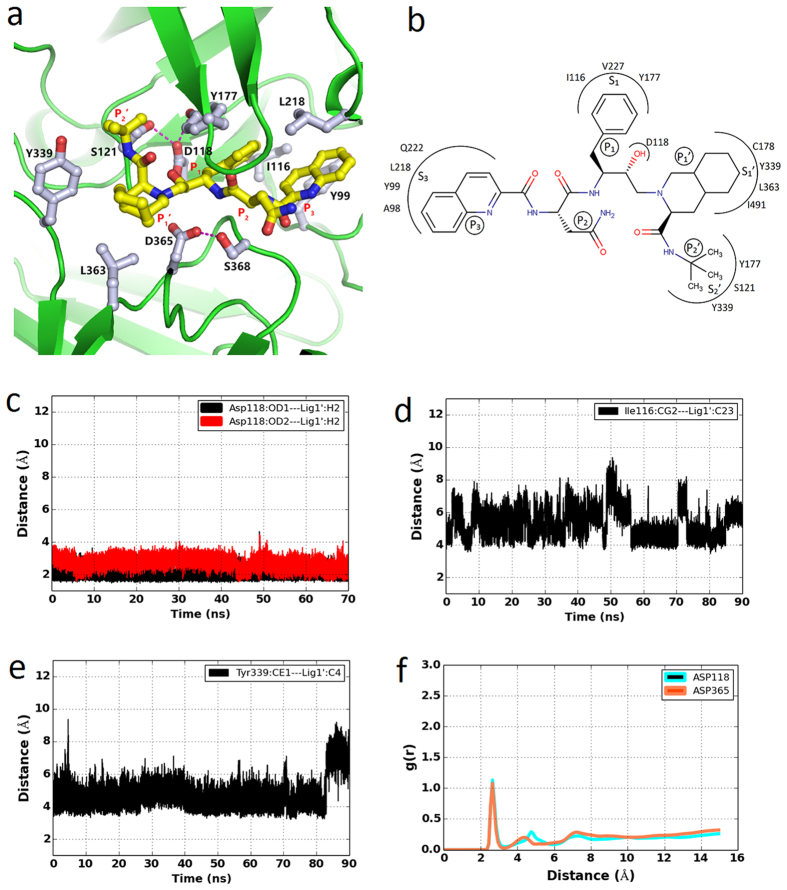
Active site of the PfPMV-saquinavir complex. (**a**) Zoomed in view of the active site. Bound saquinavir is shown as ball and stick model with carbon colored in yellow. Residues involved in saquinavir binding are shown as ball and stick with carbon colored in grey and hydrogen bonding interactions are shown as dotted lines. (**b**) Schematic representation of the saquinavir binding pocket in PfPMV. (**c**) The distance between the carboxylate oxygen atoms (OD1 and OD2) of Asp118 and central hydroxyl group hydrogen (H2) of saquinavir (Lig1) during simulation. (**d**) The distance between the side chain carbon atom (CG2) of PfPMV Ile116 and saquinavir P1 position phenyl group carbon atom (C23) during simulation. (**e**) The distance between the side chain carbon atom (CE1) of PfPMV Tyr339 and saquinavir P1′ position hydrophobic group carbon atom (C4) during simulation. (**f**) The radial distribution function [g(r)] of water hydrogen atoms around the carboxylate oxygen atoms of Asp118 and Asp365 during simulation.

Saquinavir is a potent inhibitor of HIV-1 protease and it is the first anti-HIV drug approved for AIDS treatment^50^. Biochemical data suggested that PfPMV was also partially inhibited by saquinavir^27^,^29^. Our docking result also shows that saquinavir has the best affinity compared to other inhibitors. In order to understand the molecular details of the interactions between saquinavir and PfPMV, a molecular dynamics simulation was performed. The stability of the PfPMV-saquinavir complex structure was monitored by checking for good stereochemical geometry (Fig. S2c) of the residues of the final structure as shown in the Ramachandran plot and the r.m.s.d. change (Fig. 1c,d) during the entire simulation. The change in energies of the system (Fig. S11) also reflects the stability of the simulation system during the entire simulation process. During the simulation, movements of Insert-2, 3 domains and the tip of the flap are observed (Fig. S4c). However, the core of the structure remains stable. A snapshot structure of the PfPMV-saquinavir complex with the lowest r.m.s.d. value, with respect to the average structure from the last 20 ns of the simulation trajectory, was used to analyze the critical interactions of the inhibitor with the enzyme active site residues. Analysis of the PfPMV-saquinavir complex structure shows that the directionality and mode of binding of saquinavir is similar to that of the PEXEL substrate (Figs 7a,b and S10a). Saquinavir is a peptidomimetic inhibitor designed with a hydroxyl group between the P1 and P1′ position and large hydrophobic groups in P3, P1 and P1′ positions of a peptide substrate (Fig. 7b). In the PfPMV-saquinavir complex structure, the central hydroxyl group of the inhibitor is placed between the carboxylate groups of Asp118 and Asp365 and remains hydrogen-bonded to Asp118 (Fig. 7c). The carboxylate group of Asp365 forms a hydrogen bond with the side chain of Ser368. The placement of the central hydroxyl group between two aspartates removes the catalytic water molecule (Fig. 7f) from the active site and thus inhibits the enzyme. A similar interaction of the peptidomimetic inhibitor hydroxyl group with pepsin^36^,^43^ and other vacuolar plasmepsins^39^ has been reported. Due to the placement of the central hydroxyl group of saquinavir between the Asp118 and Asp365 carboxylate groups, the degree of hydration of these two catalytic residues decreased significantly compared to the apo- and PEXEL-bound PfPMV structures. The bulky hydrophobic group at the P3 position of the inhibitor is bound to a pocket formed by the hydrophobic side chains of Tyr99, Ile116, and Leu218. The central phenyl group (P1) is bound to the hydrophobic pocket formed predominantly by Ile116 (Fig. 7d). Binding of the phenyl group of saquinavir in the hydrophobic S1 pocket may also explain the loose binding of a serine protease inhibitor PMSF in the same pocket and hence cause partial inhibition of the recombinant enzyme by this inhibitor^32^. The flap is in the partially open conformation and does not interact with the central phenyl group of saquinavir. The opening of flap as a result of the binding of the inhibitor in the active site has been previously observed^51^. The hydrophobic group at the P1′ position is placed in a pocket formed by Tyr339 and Leu363 (Fig. 7e). The –NH group of the peptide bond between the terminal trimethyl group and the P1′ hydrophobic group forms a hydrogen bond with the main chain carbonyl group of Gly120 (Fig. S10b). The trimethyl group at P2′ position interacts hydrophobically with Tyr177 from the flap region.

The comparison of PfPMV-saquinavir and PfPMV-PEXEL complexes indicates that saquinavir is bound to the PfPMV active site pocket mainly through hydrophobic interactions and a few polar interactions, whereas the PEXEL substrate is bound *via* several strong polar interactions and optimal hydrophobic interactions. In contrast to the binding of saquinavir to PfPMV, the same inhibitor is tightly bound to the HIV-1 protease by its two subunits. The binding pocket formed by the two HIV-1 protease molecules properly accommodates the hydrophobic groups of saquinavir placing the central hydroxyl group in between two catalytic aspartates^52^. In HIV-1 protease structure (PDB ID = 2NNP), saquinavir is present as two conformations. On the other hand, the structure of endothiapepsin with saquinavir (PDB ID = 3PWW) demonstrates asymmetry in the position of the central hydroxyl group towards the catalytic aspartates as in our modelled structure, with the former hydroxyl group is hydrogen bonded to Asp35 (structural analog of Asp118). Due to the asymmetry in the active sites of pepsin-like aspartic proteases, it is highly probable that the single hydrogen bond between the central hydroxyl and one of the aspartates is a feature in the binding mode of saquinavir. We believe, therefore, that saquinavir could be optimised to develop a potent inhibitor of PfPMV.

## Concluding remarks

*Plasmodium falciparum* plasmepsin V (PfPMV) is an essential protease for the survival of the parasite as this enzyme helps in exporting hundreds of proteins by cleavage at the PEXEL sequence motif (RxLxE/Q/D)^53^,^54^. We have developed a structural model of PfPMV and conducted extensive molecular dynamics simulations of the mature enzyme consisting of residues Ser82 to Leu522 as its apo form, as well as in its complexes with PEXEL substrate and with the inhibitor saquinavir. PfPMV has a core structural fold similar to that observed for pepsin-like aspartic protease family proteins. However, in the PfPMV structure there are three insertion regions. Two of the insertion domains (Insert-2 and 3) have helix-turn-helix motifs. The secondary structural elements and charge distribution on the surface of these two insertion domains indicate that they might be involved in interactions with endoplasmic reticulum membrane as has been observed for plant specific saposin-like aspartic protease insertion domains^38^. The flap is flexible in the apo- structure and adopts a closed conformation in the PEXEL-bound complex. The results from docking and molecular dynamics simulation studies show that the specificity of arginine at the P3 position of PEXEL substrate is mainly due to the presence of Glu179 and Gln222. The spatial arrangement and distribution of water molecules around Asp118 and Asp365 suggests that these residues are involved in catalysis. The presence of more water molecules around the Asp365 carboxylate group in the PfPMV-PEXEL complex indicates its involvement in nucleophile generation from water molecules during catalysis similar to that observed in other pepsin-like aspartic proteases^36^. The screening of inhibitors using docking studies has proven that presence of phenyl group at the P1 position of the inhibitor results in better binding at the PfPMV active site. The docking results and molecular dynamics simulation of the PfPMV-saquinavir complex agree with the experimentally-proven inhibition of this enzyme by retroviral protease inhibitors^7^,^27^,^32^ and KNI inhibitors^30^. Structural analysis of PfPMV-saquinavir complex structure reveals that the inhibition by retroviral protease inhibitors and KNI inhibitors may not only result from the replacement by catalytic water between Asp118 and Asp365, but may also result from the restriction of flap closure. Hence, our structural model of PfPMV and molecular dynamics simulation studies provide a detailed insight into the active site and substrate binding pocket of this enzyme. HIV-1 protease inhibitors and KNI compounds should be explored for development of potent PfPMV inhibitors that can be used as antimalarial drugs.

## Methods

### Homology modeling of PfPMV structure

The protein sequence of *P. falciparum* plasmepsin V (PfPMV) was retrieved from NCBI nucleotide database (accession no: AAW71468.1). The zymogen and the mature domain of the protein were predicted after sequence alignment of the mature vacuolar plasmpesins (PMI, PMII, HAP and PMIV) with that of PfPMV sequence. The predicted N-terminal signal sequence (Met1-Val64) and C-terminal membrane anchoring region (Thr523-Thr590) were deleted before submitting the sequence for homology modeling. The structure of PfPMV zymogen was predicted by the I-TASSER server^35^, which is an automated protein structure prediction server reported to be the best in the recent community-wide Critical Assessment of protein Structure Prediction experiments (CASP)^55^. The truncated PfPMV zymogen sequence (Glu65-Leu522) was submitted to the I-TASSER server and no structure template was specified by us during the submission. The server automatically chose the template structures based on aligning the query PfPMV sequence with the sequences of vacuolar plasmepsins, pepsinogens, cathepsins and *P. vivax* plasmepsin V. The structure templates used by I-TASSER for automated homology model generation after sequence alignment are: Plasmepsin II (1XDH, sequence identity 17.0%), Cathepsin (1TZS, sequence identity 22.0%), Pepsinogen (2PSG, sequence identity 19.3%), BACE-2 (2EWY, sequence identity 21.8%) and PvPMV (4ZL4, sequence identity 60.0%). Based on the sequence alignment and available structures of similar proteins, the homology model of PfPMV was built by I-TASSER. The best model was identified based on the C-score (−0.41) as calculated from the relative clustering structural density and consensus significance. In addition, other parameters such as TM score (0.66 ± 0.13), cluster density (0.1387) were also used to evaluate the estimate accuracy of the best model. The best model of PfPMV was analyzed using COOT^56^ and Pymol^57^. The interactions in the active of the PfPMV-PEXEL and PfPMV-saquinavir complexes were analyzed by Ligplot^58^. The mature PfPMV (Ser82-Leu522) structure used in this study was generated by deleting the first 17 residues of the prosegment.

### Docking of inhibitors in the PfPMV active site

Inhibitors/ligands bound to aspartic proteases from Protein Data Bank (PDB, www.rcsb.org) and a few compounds were selected from the literature. The structural coordinates of most of the ligands were taken from PDB since their crystal structures as a complex were available. The structures of few selected ligands were prepared by the PRODRG2^59^ server as crystal structures of those ligands were not available in the PDB. The mature PfPMV structure (Ser82-Leu522) and ligand structures were converted to pdbqt format, using the MGL (Molecular Graphics Laboratory) tools 1.5.4^60^. Polar hydrogens and gasteiger charges were added to both protein and ligand structures for converting the coordinates to pdbqt file and these converted structure files (.pdbqt) were used for docking using Autodock Vina 1.1.2^61^. The grid spacing was changed to 1.0 Å and a grid box with dimensions 22 × 22 × 22 Å^3^ was defined centred at a point between carboxylic acid groups of Asp118 and Asp365. Other parameters were kept as default. Docking experiments were performed using the exhaustiveness value of 30. Each run resulted in nine different ligand conformations. Output results of docking runs were visualized and analysed using MGL tools 1.5.4. The ligand conformations with the highest binding affinity were selected as the best possible binding mode of that ligand in the PfPMV active site.

### Molecular dynamics simulation and analysis of the structures

The mature PfPMV structure consists of residues Ser82-Leu522. The three PfPMV structures used in this study are the apo-PfPMV, the PfPMV-PEXEL complex and the PfPMV-saquinavir complex. All-atom-molecular simulations were performed for three structures. Topology and forcefield parameters for all atoms were assigned from the CHARMM22 parameter set with the CMAP correction^62^,^63^,^64^. For saquinavir, the force field parameters were obtained from swissparam^65^. The protein, or protein–ligand complex, was first solvated using 27524 TIP3P water molecules within a rectangular box with dimensions 106 × 90 × 100 Å^3^. 80 Na^+^ and 78 Cl^−^ were added to neutralize the system and to mimic a physiological ionic concentration of 0.15 M. Long range electrostatic interactions were calculated using Particle Mesh Ewald (PME) method^66^. The cut-off for both electrostatic interactions and Vander Waals interactions was set to 12 Å. Bonds to hydrogen atoms were constrained using the SHAKE algorithm^67^. The equation of motion was integrated every 2 fs and snapshots were saved every 1 ps. The temperature was controlled using Langevin Dynamics with a friction coefficient of 1 ps^−1^. The water molecules and ions present in the system were first subjected to an energy minimization for 10000 steps with strong restraints on protein (500 kcal.mol^−1^.Å^−2^). This was followed by minimization of the entire system without any restraints for another 10000 steps. The system was then gradually heated to 298 K in 14 steps of 2 ps each with constant volume (NVT ensemble). Weak restraints of 10 kcal.mol^−1^.Å^−2^ were applied on the protein/protein–ligand complex. The density of the system was then relaxed for 40 ps by switching to an NPT ensemble with an equilibrium pressure of 1 atm and temperature of 298 K. Constant pressure was maintained using Nosé–Hoover Langevin piston pressure control^68^,^69^. The restraints on the protein were then removed and the system was further equilibrated for 100 ps. Finally, production simulations of 90 ns were carried out for each system in the NPT ensemble at 298 K and 1 atm pressure. All simulations were performed using NAMD v2.9^70^.

The qualities of the final structures after 90 ns simulation were assessed by analyzing the stereochemical geometries of the residues on the Ramachandran map. In order to obtain the most probable representative of the structures, the average structures were extracted from the entire simulation (90 ns) as well as the last 20 ns (70–90 ns) of each simulation trajectory. The average structures of PfPMV from entire simulation were analyzed for assignment of the secondary structures and domains of the mature enzyme. In order to analyze the active sites, the snapshot structures were chosen based on the lowest r.m.s.d. value, with respect to the respective average structure generated from the last 20 ns of the simulation trajectories. These snapshots structures were obtained at around 77^th^ ns (r.m.s.d. = 0.38 Å), 77^th^ ns (r.m.s.d. = 0.41 Å) and 80^th^ ns (r.m.s.d. = 0.44 Å) for apo-PfPMV, PfPMV-PEXEL and PfPMV-saquinavir, respectively. Both distance (<3.0 Å) and angle (>135°) criteria were used for identification of the hydrogen bonds, as well as for calculation of the percentage of time they exist during simulation.

## Additional Information

**How to cite this article**: Bedi, R. K. *et al*. Understanding the structural basis of substrate recognition by *Plasmodium falciparum* plasmepsin V to aid in the design of potent inhibitors. *Sci. Rep.* **6**, 31420; doi: 10.1038/srep31420 (2016).

## Supplementary Material

Supplementary Information

## Figures and Tables

**Table 1:** Inhibitors docked in the PfPMV active site and their affinity values.

Sr No	PDB ID (Name)	Binding affinity (kcal/mol)
1	ROC (Saquinavir)	−12.8
2	006 (KNI10006)	−12.2
3	JE2 (KNI764)	−11.7
4	K95 (KNI10395)	−11.7
5	74A (SCH743813)	−11.4
6	PB8	−11.4
7	BJC (BJC060)	−11
8	ZY0	−11
9	SC6 (SCH745966)	−10.7
10	VG5	−10.7
11	009	−10.7
12	1IN	−10.7
13	3HF (BMS655295)	−10.6
14	BAV (NVPBAV544)	−10.5
15	Z75 (SCH743641)	−10.4
16	AB1 (Lopinavir)	−10.4
17	PB7	−10.3
18	318 (SCH726222)	−10.3
19	VG6	−10.3
20	012	−10.3
21	PI5	−10.3
22	PI7	−10.3
23	L2T	−10.3
24	314	−10.2
25	ZYE	−10.2
26	PB0	−10.1
27	197	−10.1
28	MK1	−10.1
29	853	−10.1
30	LA1	−10.1
31	CS9	−10
32	F2I	−10
33	3HF	−10
34	JDC	−10
35	VG4	−10
36	RIT (Ritonavir)	−10
37	1UN (Nelfinavir)	−9.9
38	VG7	−9.8
39	842	−9.8
40	316	−9.7
41	MR0	−9.7
42	PI4	−9.7
43	(RVIstatin)	−9.7
44	CS5	−9.6
45	X22	−9.6
46	04C	−9.5
47	1BH	−9.5
48	SC7	−9.4
49	VG0	−9.3
50	1LI	−9.3
51	X23	−9.3
52	L0I	−9.2
53	51U	−9.2
54	C20	−9.2
55	(KVLstatin)	−9.1
56	(RVLstatin)	−9
57	TZT	−8.8
58	DFK	−8.7
59	(Pepstatin)	−8.7
60	A49	−8.3
61	FR1	−8.2
62	K7J	−8.1
63	X93	−8
64	EAL	−7.7
65	KHA	−7.6
