# Supplementary material for: Understanding the structural basis of substrate recognition by Plasmodium falciparum plasmepsin V to aid in the design of potent inhibitors
Source: Sci Rep. 2016 Aug 17;6:31420. doi: 10.1038/srep31420 (PMC4987639; doi:10.1038/srep31420)
Supplement: Supplementary Information [file srep31420-s1.pdf]

Understanding the structural basis of substrate recognition by *Plasmodium falciparum* plasmepsin V to aid in the design of potent inhibitors

Rajiv K. Bedi[1+], Chandan Patel[2+], Vandana Mishra[1], Huogen Xiao[3], Rickey Y. Yada[4], Prasenjit Bhaumik[1*]

[1]Department of Biosciences and Bioengineering, Indian Institute of Technology Bombay, Powai, Mumbai - 400076, India

[2]Department of Chemistry, Indian Institute of Technology Bombay, Powai, Mumbai - 400076, India

[3]Department of Molecular and Cellular Biology, University of Guelph, Guelph, Ontario, N1G 2W1, Canada

[4]Faculty of Land and Food Systems, University of British Columbia, 248-2357 Main Mall, Vancouver, BC V6T 1Z4, Canada.

[*]Corresponding author E-mail: pbhaumik@iitb.ac.in

[+] These authors contributed equally to this work.

Supporting information

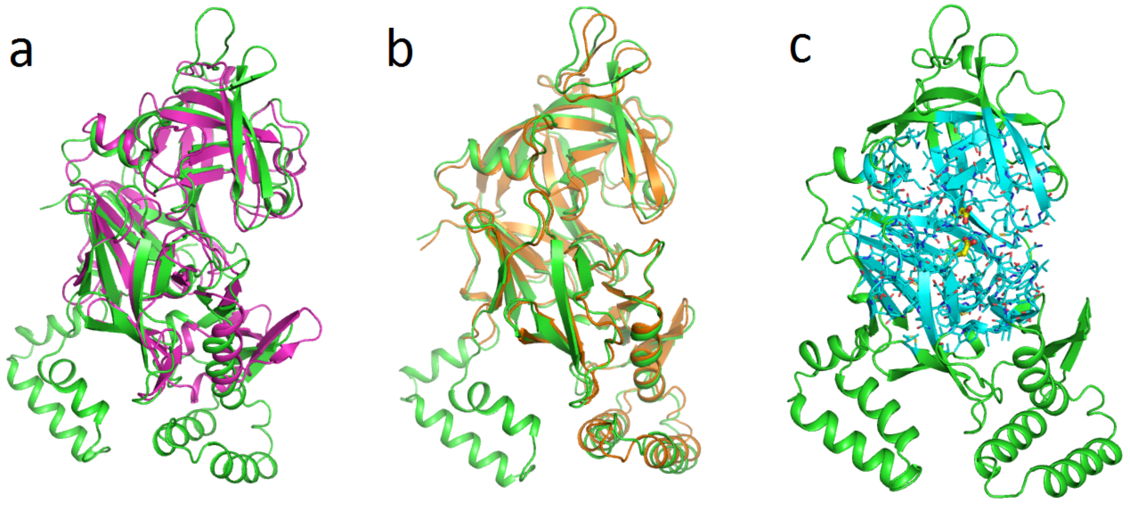

S1 Figure: Structural fold of mature PfPMV and its comparasion. (**a**) Superimposition of PfPMV structure (green) with pepsin structure (magenta). (**b**) Superimposition of PfPMV structure (green) with PvPMV structure (orange). Insert-2 region is missing in the crystal structure of PvPMV. (**c**) The residues within 15 Å radius of the active site aspartates (ball and stick) are considered as PfPMV structural core and shown as lines with cyan color carbon.

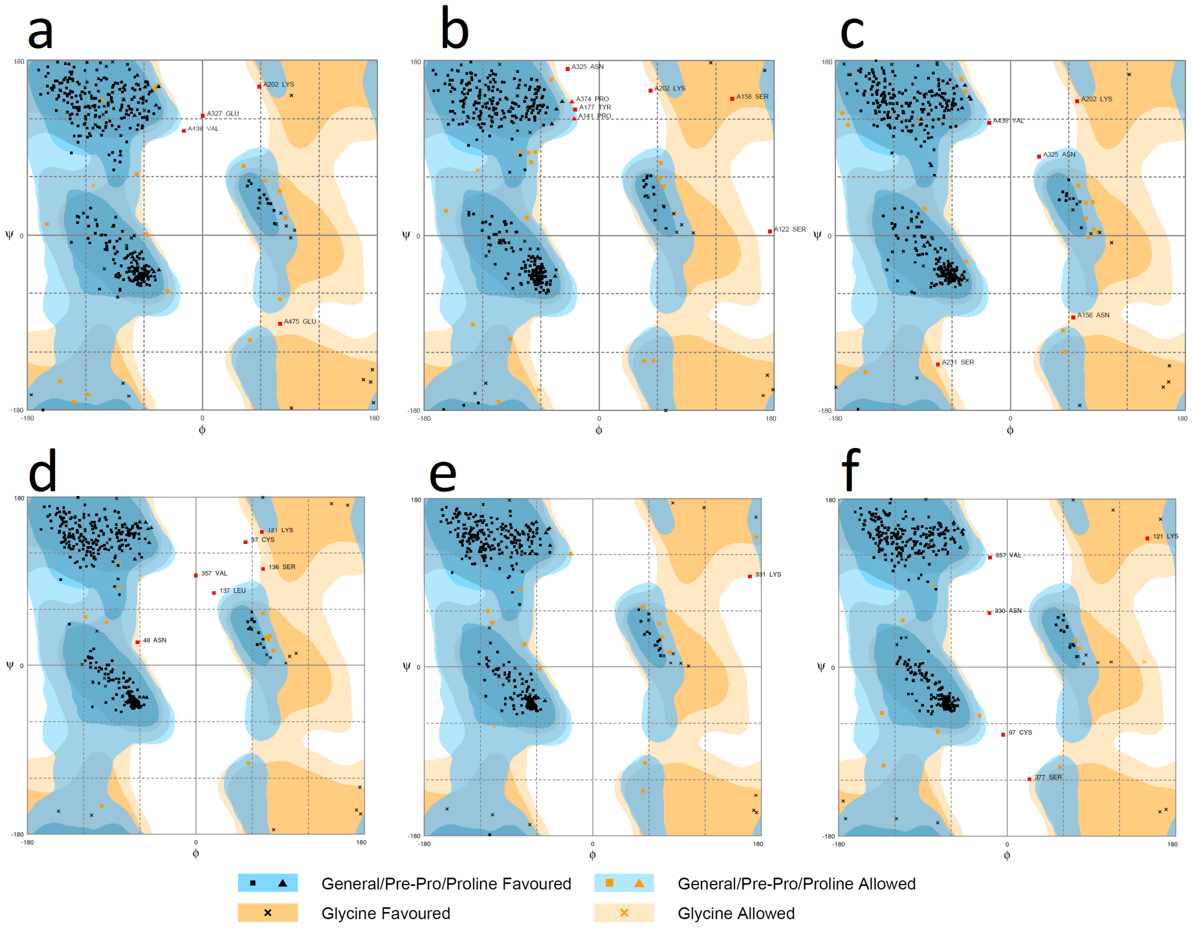

S2 Figure: Stereochemical geometry of different forms of PfPMV structures. Ramachandran plot of the final (**a**) apo-PfPMV, (**b**) PfPMV-PEXEL complex and (**c**) PfPMV-saquinavir complex. Ramachandran plot of the sampled average (**d**) apo-PfPMV, (**e**) PfPMV-PEXEL complex and (**f**) PfPMV-saquinavir complex. Residues in outlier region are marked as red squares. The plots were made using RAMPAGE[1].

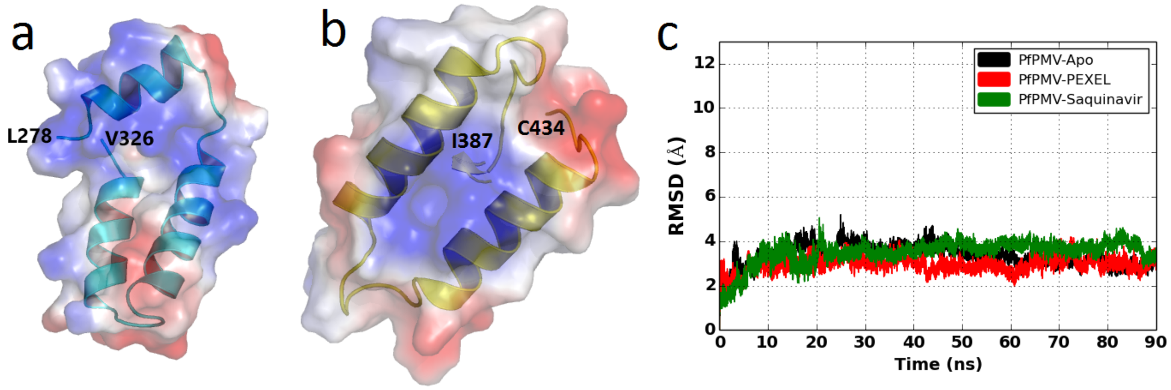

S3 Figure: Insert-2 and Insert-3 regions in PfPMV structure. The surface diagrams showing the electrostatic charge distribution on the Insert-2 (**a**) and Insert-3 (**b**) regions. The positive , negative and neutral charged surfaces are represented with blue, red and light grey colors, respectively. The secondary structural elements are shown as cartoon inside the surface. (**c**) Plot of r.m.s.d. change for Insert-2 regions of apo-PfPMV (black), PfPMV-PEXEL complex (red) and PfPMV-saquinavir complex (green) during the simulation.

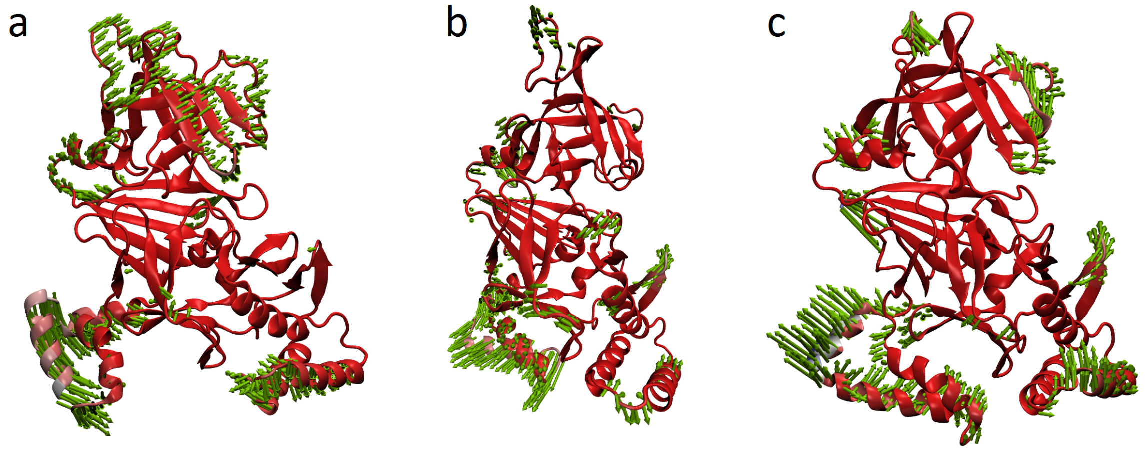

S4 Figure: First principal component of the protein for the simulation trajectory. (**a**) apo-PfPMV, (**b**) PfPMV-PEXEL complex, and (**c**) PfPMV-saquinavir complex. Only components showing deviation of more than 2 Å are shown. Protein is colored using RGB scale centered around 0.1. The residues in red and blue show minimum and maximum movement, respectively. Green arrows show the direction of motion.

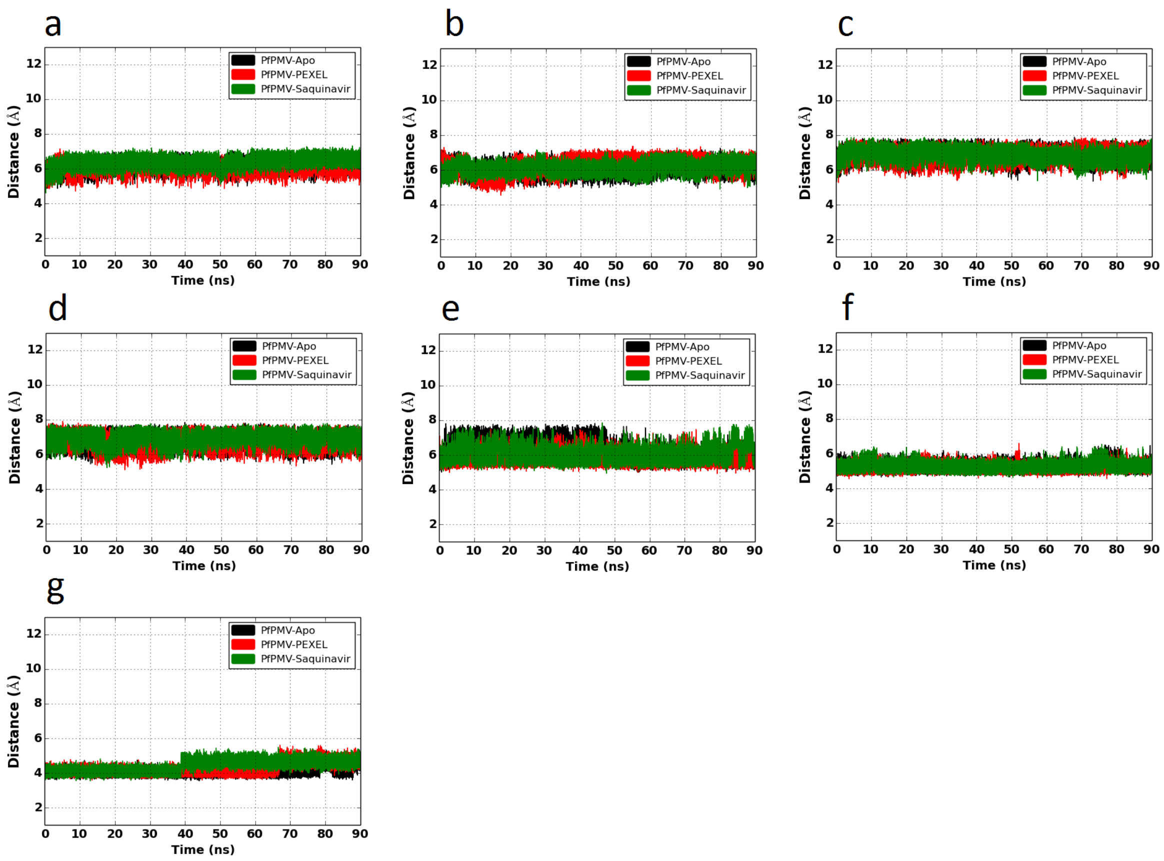

S5 Figure: Change in distance between the Cα atoms of two cysteine residues involved in forming disulfide bonds in apo-PfPMV (black), PfPMV-PEXEL complex (red) and PfPMV-saquinavir complex (green) during the simulation. (**a**) Cys128-Cys211, (**b**) Cys131-Cys134, (**c**) Cys155-Cys166, (**d**) Cys160-Cys171, (**e**) Cys259-Cys518, (**f**) Cys389-Cys434 and (**g**) Cys443-Cys479.

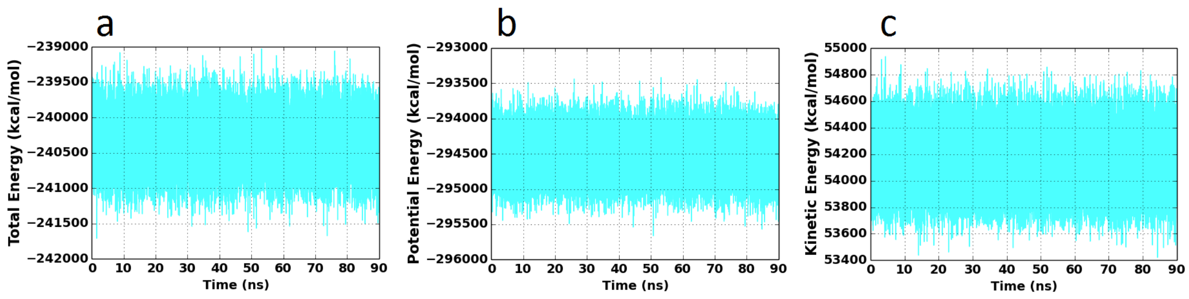

S6 Figure: Variation of energies of the apo-PfPMV simulation system. (**a**) Total energy, (**b**) Potential energy and (**c**) Kinetic energy.

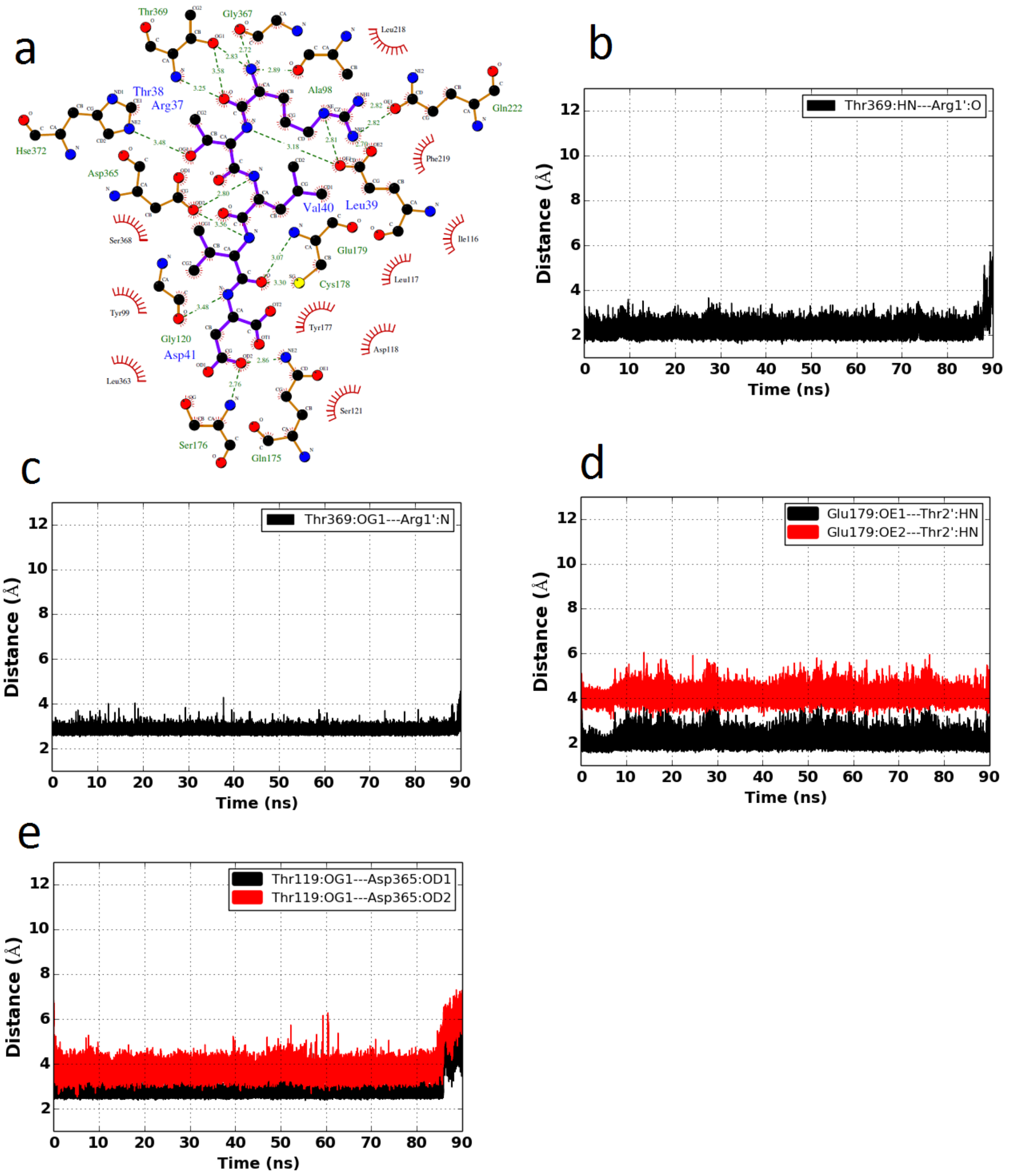

S7 Figure: Interactions between PEXEL substrate and PfPMV active site residues. (**a**) Schematic diagram (prepared using Ligplot) showing the interactions of PEXEL substrate with PfPMV active site residues. The PEXEL substrate bonds are shown in purple and the bonds in PfPMV are shown in orange. Hydrogen bonds are green dashed lines with distance in Å. Hydrophobic contacts with protein are presented by red semicircles with radiating lines. (**b**) The distance between carbonyl oxygen atom (O) of PEXEL arginine and main chain -NH

group of PfPMV Thr369. (**c**) The distance between amino terminal nitrogen atom (N) of PEXEL arginine and side chain hydroxyl group oxygen (OG1) of PfPMV Thr369. (**d**) The distance between the main chain -NH group hydrogen atom (NH) of PEXEL threonine and carboxylate oxygen atoms (OE1 and OE2) of PfPMV Glu179. (**e**) The distance between Asp365 carboxylate oxygen atoms (OD1 and OD2) and Thr119 side chain hydroxyl oxygen atom (OG1) of PEXEL.

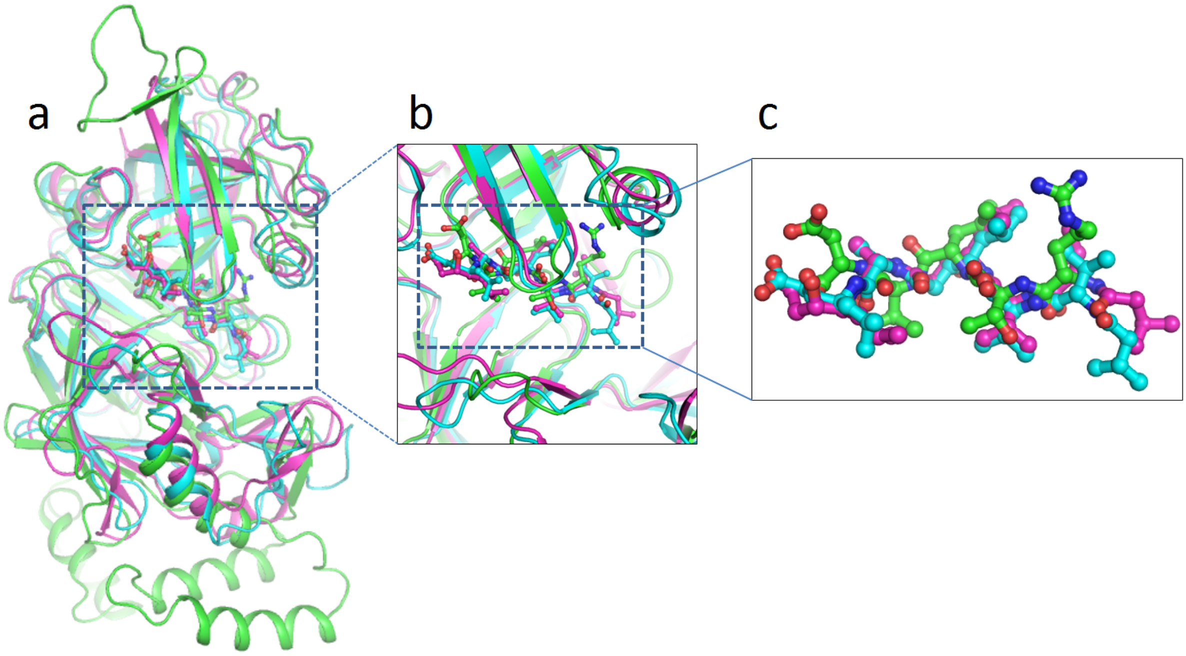

S8 Figure: Comparison of mode of PEXEL substrate binding in PfPMV to the mode of peptidomimetic inhibitor binding in other pepsin-like aspartic proteases. (**a**) Structural superposition of PfPMV-PEXEL complex (green) with PMII-pepstatin complex (cyan), pepsin-pepstatin complex (magenta). The PEXEL substrate and pepstatin are shown as ball and stick model. (**b**) Zoomed in view of the substrate or inhibitor binding pocket presented in panel a. (**c**) Zoomed in view of the mode of binding of PEXEL in PfPMV, pepstatin in PMII and pepsin active sites.

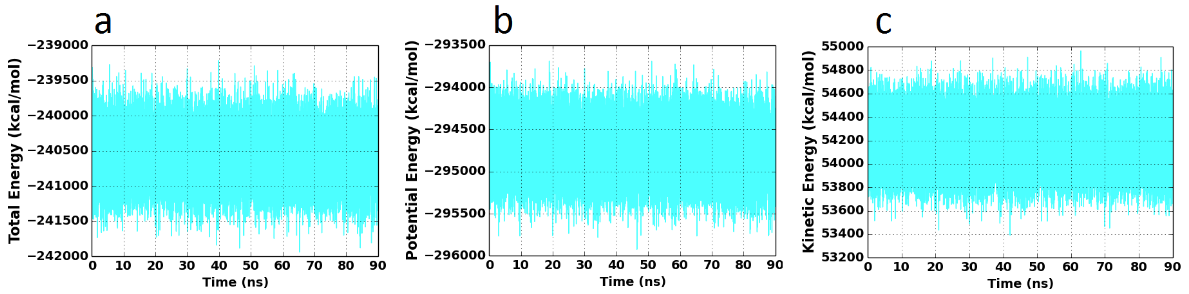

S9 Figure: Variation of energies of the PfPMV-PEXEL complex simulation system. (**a**) Total energy, (**b**) Potential energy and (**c**) Kinetic energy.

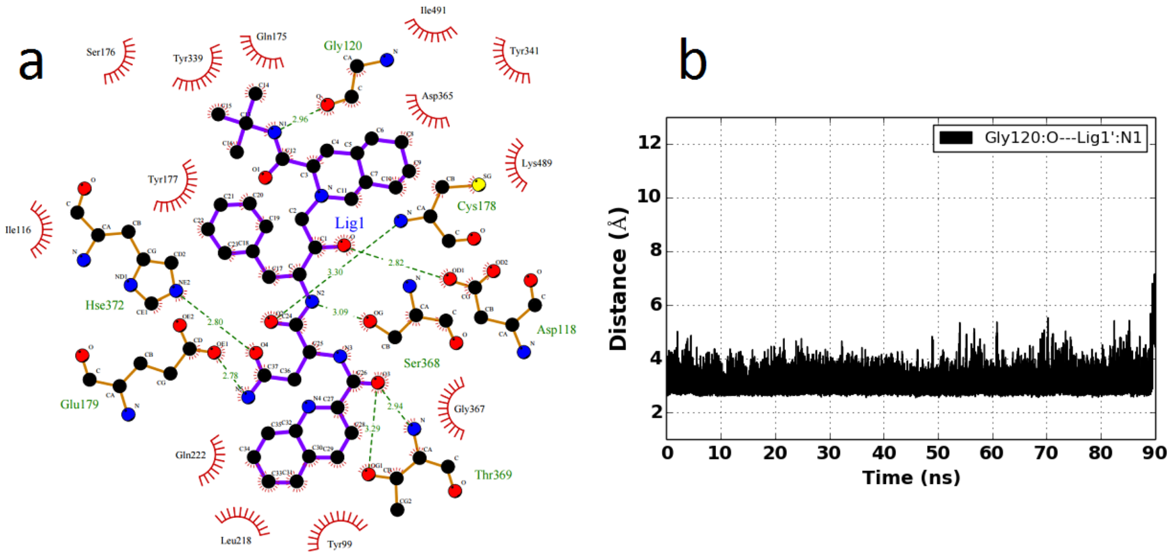

S10 Figure: Interactions of saquinavir in the PfPMV substrate binding pocket. (**a**) Schematic diagram (prepared using Ligplot) showing the interactions of saquinavir**.** Saquinavir bonds are shown in purple and the bonds in PfPMV are shown in orange. Hydrogen bonds are green dashed lines with distance in Å. Hydrophobic contacts with protein are presented by red semicircles with radiating lines. (**b**) Distance between the side chain carbonyl oxygen atom (O) of PfPMV Gly120 and the nitrogen atom of the -NH group of the peptide bond between the terminal trimethylgroup and P1' hydrophobic group of saquinavir during simulation.

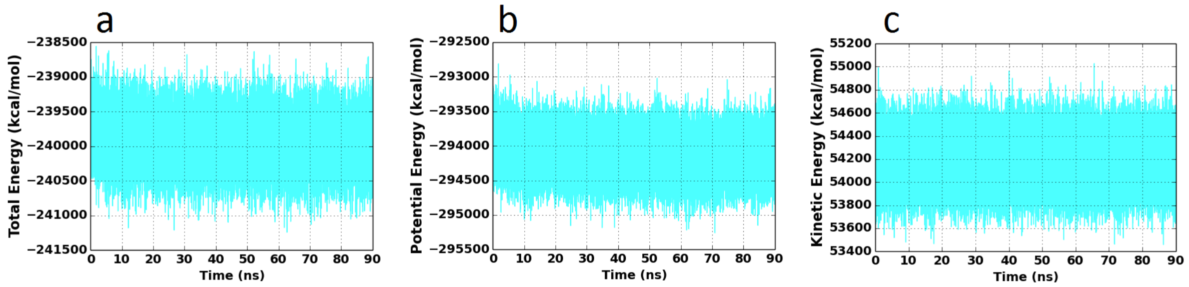

S11 Figure: Variation of energies of the PfPMV-saquinavir complex simulation system.

(**a**) Total energy, (**b**) Potential energy and (**c**) Kinetic energy.

Sr No	PDB ID (Name) Reference	Structure
1	ROC (Saquinavir)[2]	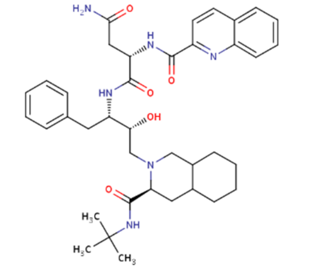
2	006 (KNI10006)[3]	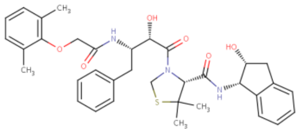
3	JE2 (KNI764)[4]	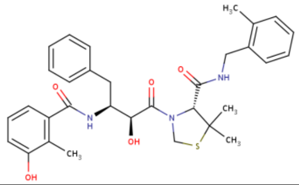
4	K95 (KNI10395)[5]	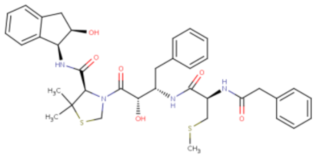
5	74A (SCH743813) [6]	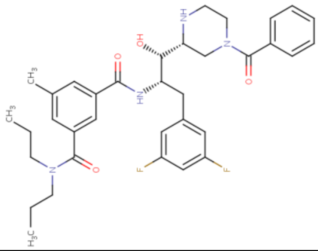
6	PB8[7]	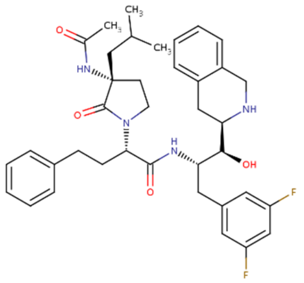
7	BJC (BJC060)[8]	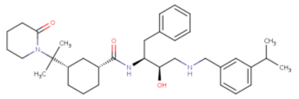
8	ZY0[9]	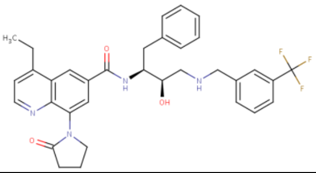
9	SC6 (SCH745966) [10]	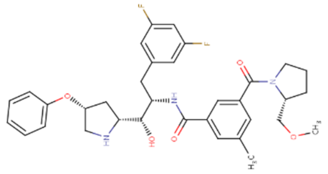
10	VG5[11]	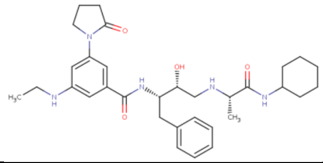
11	009[12]	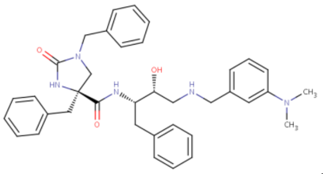
12	1IN[13]	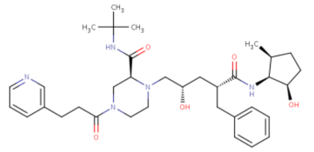
13	3HF (BMS655295) [14]	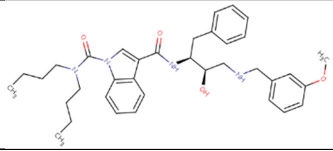
14	BAV (NVPBAV54 4)[15]	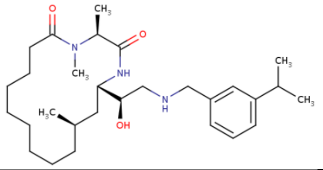
15	Z75 (SCH743641) [6]	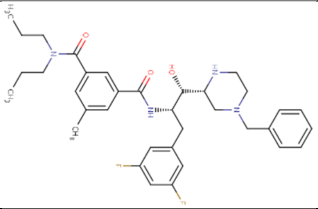
16	AB1 (Lopinavir)[16]	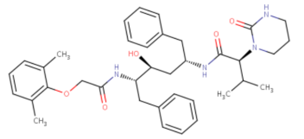
17	PB7[7]	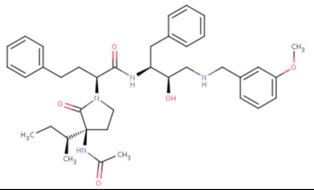
18	318 (SCH726222) [17]	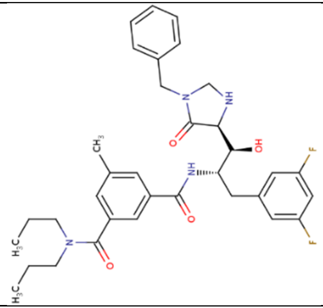

19	VG6[18]	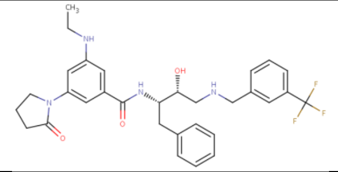
20	012[12]	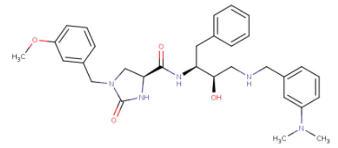
21	PI5[19]	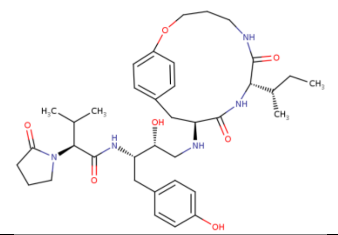
22	PI7[19]	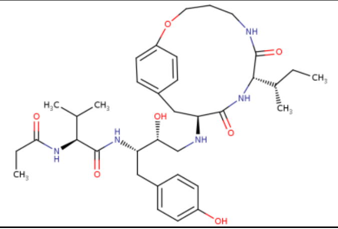
23	L2T[20]	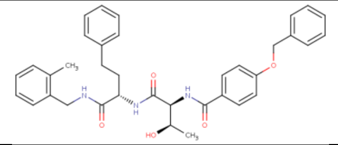
24	314[17]	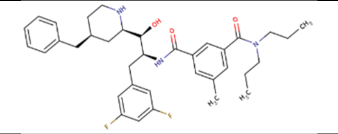
25	ZYE[9]	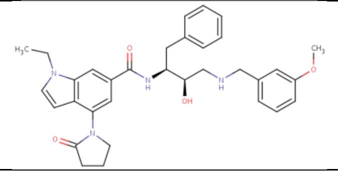
26	PB0[21]	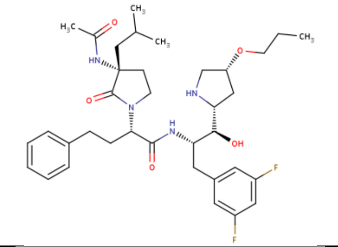
27	197[22]	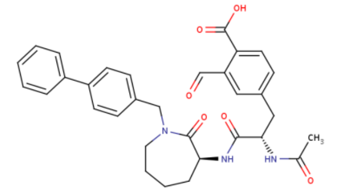
28	MK1[13]	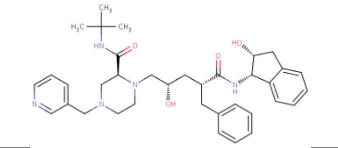
29	853[22]	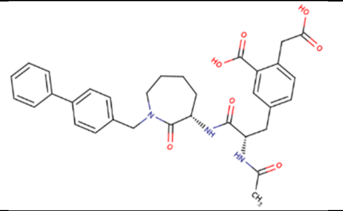
30	LA1[23]	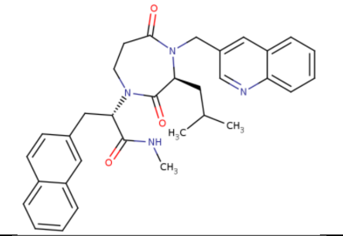
31	CS9[24]	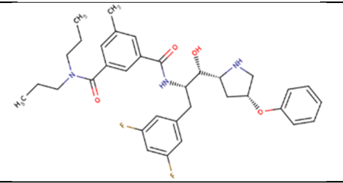
32	F21[25]	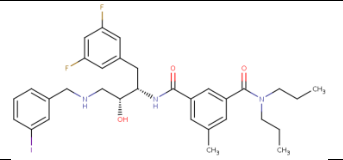
33	3HF[14]	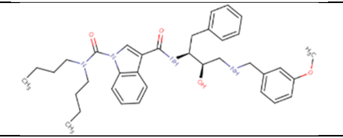
34	JDC[26]	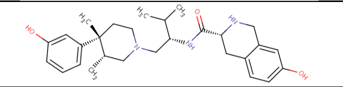
35	VG4[11]	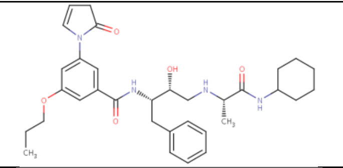
36	RIT (Ritonavir)[27]	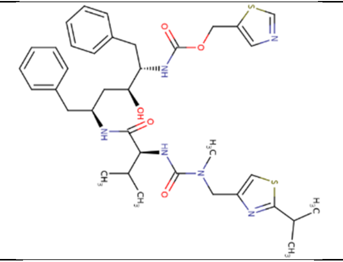
37	1UN (Nelfinavir)[28]	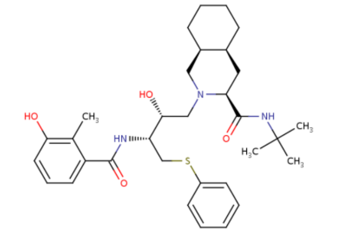
38	VG7[18]	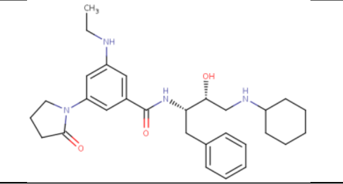

39	842[29]	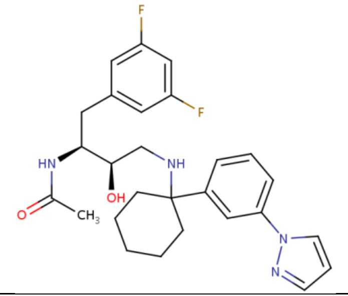
40	316[17]	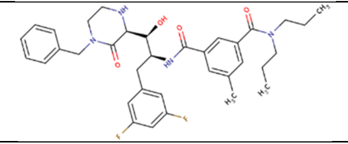
41	MR0[30]	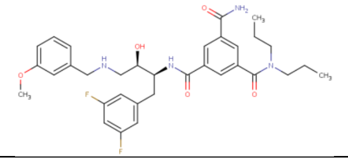
42	PI4[19]	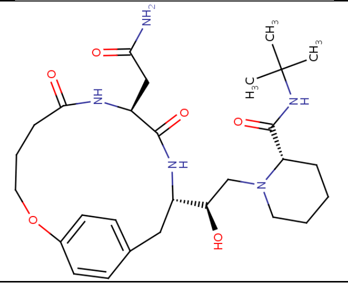
43	RVIstatin[31]	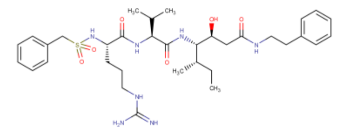
44	CS5[32]	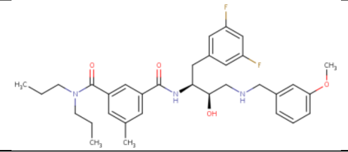
45	X22[33]	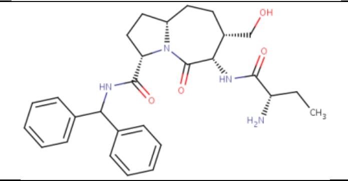
46	04C[34]	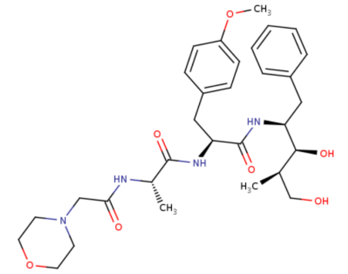
47	1BH[35]	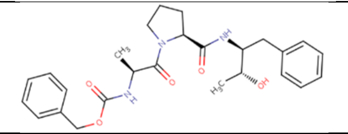

48	SC7[24]	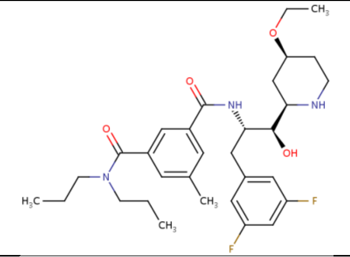
49	VG0[18]	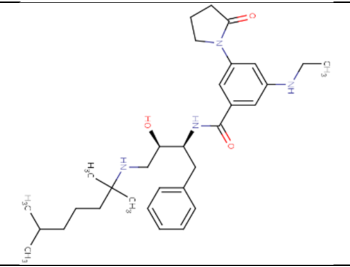
50	1LI[36]	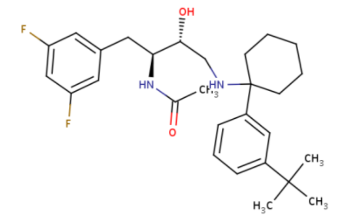
51	X23[33]	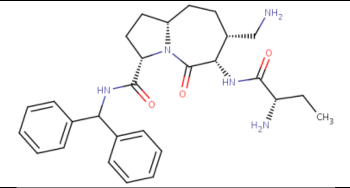
52	L0I[37]	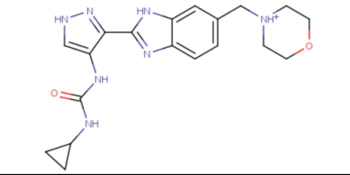
53	51U (To be Published)	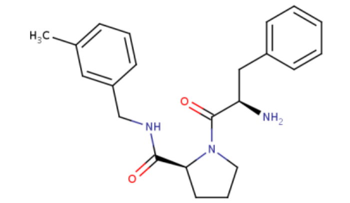
54	C20[38]	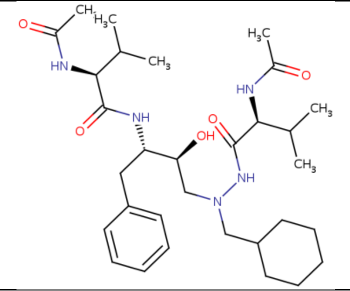
55	KVLstatin[31]	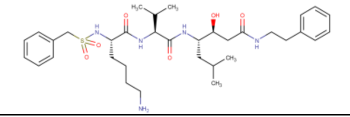
56	RVLstatin[31]	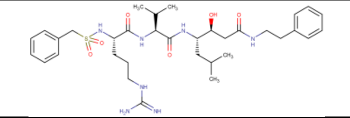

57	TZT[39]	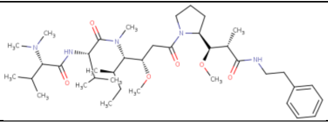
58	DFK[40]	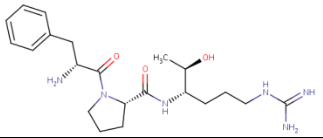
59	Pepstatin A[41]	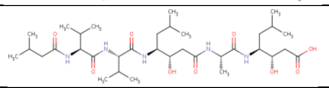
60	A49[42]	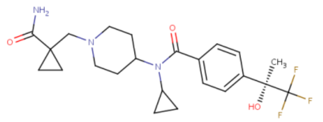
61	FR1[43]	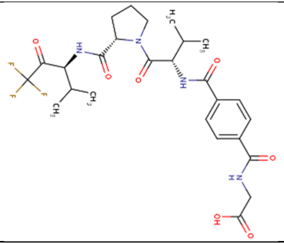

62	K7J[44]	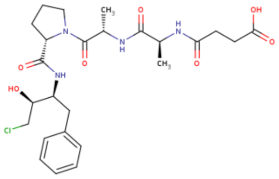
63	X93[45]	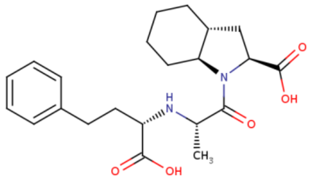
64	EAL[46]	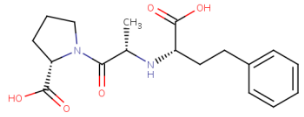
65	KHA[47]	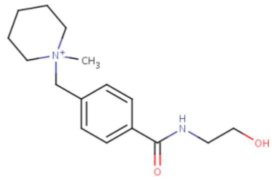

S1 Table: List of inhibitors docked in PfPMV active site. The covalent structures of the inhibitors are also presented with their PDB IDs. The references for the ligands are in superscript.

References

1 Lovell, S. C. *et al.* Structure validation by Cα geometry: ϕ, ψ and Cβ deviation. *Proteins* **50**, 437-450 (2003). URL: http://mordred.bioc.cam.ac.uk/~rapper/rampage.php

2 Tie, Y. *et al.* Atomic resolution crystal structures of HIV-1 protease and mutants V82A and I84V with saquinavir. *Proteins* **67**, 232-242 (2007).

3 Bhaumik, P. *et al.* Crystal structures of the histo-aspartic protease (HAP) from *Plasmodium falciparum*. *J. Mol. Biol.* **388**, 520-540 (2009).

4 Clemente, J. C. *et al.* Structure of the aspartic protease plasmepsin 4 from the malarial parasite *Plasmodium malariae* bound to an allophenylnorstatine-based inhibitor. *Acta Crystallogr. D Biol. Crystallogr.* **62**, 246-252 (2006).

5 Bhaumik, P. *et al.* Structural insights into the activation and inhibition of histo-aspartic protease from *Plasmodium falciparum*. *Biochemistry* **50**, 8862-8879 (2011).

6 Cumming, J. *et al.* Piperazine sulfonamide BACE1 inhibitors: design, synthesis, and in vivo characterization. *Bioorg. Med. Chem. Lett.* **20**, 2837-2842 (2010).

7 Thompson, L. A. *et al.* Synthesis and in vivo evaluation of cyclic diaminopropane BACE-1 inhibitors. *Bioorg. Med. Chem. Lett.* **21**, 6909-6915 (2011).

8 Hanessian, S. *et al.* Structure-based design and synthesis of novel P2/P3 modified, non-peptidic β-secretase (BACE-1) inhibitors. *Bioorg. Med. Chem. Lett.* **20**, 1924-1927 (2010).

9 Charrier, N. *et al.* Second generation of BACE-1 inhibitors. Part 1: The need for improved pharmacokinetics. *Bioorg. Med. Chem. Lett.* **19**, 3664-3668 (2009).

10 Iserloh, U. *et al.* Discovery of an orally efficaceous 4-phenoxypyrrolidine-based BACE-1 inhibitor. *Bioorg. Med. Chem. Lett.* **18**, 418-422 (2008).

11 Clarke, B. *et al.* BACE-1 inhibitors part 1: identification of novel hydroxy ethylamines (HEAs). *Bioorg. Med. Chem. Lett.* **18**, 1011-1016 (2008).

12 Park, H. *et al.* Synthesis, SAR, and X-ray structure of human BACE-1 inhibitors with cyclic urea derivatives. *Bioorg. Med. Chem. Lett.* **18**, 2900-2904 (2008).

13 Munshi, S. *et al.* Rapid X-ray diffraction analysis of HIV-1 protease–inhibitor complexes: inhibitor exchange in single crystals of the bound enzyme. *Acta Crystallogr. D Biol. Crystallogr.* **54**, 1053-1060 (1998).

14 Marcin, L. R. *et al.* Synthesis and SAR of indole-and 7-azaindole-1, 3-dicarboxamide hydroxyethylamine inhibitors of BACE-1. *Bioorg. Med. Chem. Lett.* **21**, 537-541 (2011).

15 Machauer, R. *et al.* Macrocyclic peptidomimetic β-secretase (BACE-1) inhibitors with activity in vivo. *Bioorg. Med. Chem. Lett.* **19**, 1366-1370 (2009).

16 Lin, Y.-C. *et al.* Structural basis for drug and substrate specificity exhibited by FIV encoding a chimeric FIV/HIV protease. *Acta Crystallogr. D Biol. Crystallogr.* **67**, 540-548 (2011).

17 Cumming, J. *et al.* Rational design of novel, potent piperazinone and imidazolidinone BACE1 inhibitors. *Bioorg. Med. Chem. Lett.* **18**, 3236-3241 (2008).

18 Clarke, B. *et al.* BACE-1 inhibitors part 2: identification of hydroxy ethylamines (HEAs) with reduced peptidic character. *Bioorg. Med. Chem. Lett.* **18**, 1017-1021 (2008).

19 Martin, J. L. *et al.* Molecular recognition of macrocyclic peptidomimetic inhibitors by HIV-1 protease. *Biochemistry* **38**, 7978-7988 (1999).

20 Blackburn, C. *et al.* Characterization of a new series of non-covalent proteasome inhibitors with exquisite potency and selectivity for the 20S beta5-subunit. *Biochem. J.* **430**, 461-476, (2010).

21 Boy, K. M. *et al.* Monosubstituted γ-lactam and conformationally constrained 1, 3-diaminopropan-2-ol transition-state isostere inhibitors of β-secretase (BACE). *Bioorg. Med. Chem. Lett.* **21**, 6916-6924 (2011).

22 Lange, G. *et al.* Requirements for specific binding of low affinity inhibitor fragments to the SH2 domain of pp60Src are identical to those for high affinity binding of full length inhibitors. *J. Med. Chem.* **46**, 5184-5195 (2003).

23 Wattanasin, S. *et al.* 1, 4-Diazepane-2, 5-diones as novel inhibitors of LFA-1. *Bioorg. Med. Chem. Lett.* **15**, 1217-1220 (2005).

24 Iserloh, U. *et al.* Potent pyrrolidine-and piperidine-based BACE-1 inhibitors. *Bioorg. Med. Chem. Lett.* **18**, 414-417 (2008).

25 Maillard, M. C. *et al.* Design, synthesis, and crystal structure of hydroxyethyl secondary amine-based peptidomimetic inhibitors of human β-secretase. *J. Med. Chem.* **50**, 776-781 (2007).

26 Wu, H. *et al.* Structure of the human [kgr]-opioid receptor in complex with JDTic. *Nature* **485**, 327-332 (2012).

27 Sevrioukova, I. F. & Poulos, T. L. Structure and mechanism of the complex between cytochrome P4503A4 and ritonavir. *Proc. Natl. Acad. Sci.* **107**, 18422-18427 (2010).

28 King, N. M. *et al.* Extreme Entropy–Enthalpy Compensation in a Drug-Resistant Variant of HIV-1 Protease. *ACS Chem. Biol.* **7**, 1536-1546 (2012).

29 Truong, A. P. *et al.* Improving the permeability of the hydroxyethylamine BACE-1 inhibitors: Structure–activity relationship of P2′ substituents. *Bioorg. Med. Chem. Lett.* **20**, 4789-4794 (2010).

30 Kortum, S. W. *et al.* Potent and selective isophthalamide S 2 hydroxyethylamine inhibitors of BACE1. *Bioorg. Med. Chem. Lett.* **17**, 3378-3383 (2007).

31 Sleebs, B. E. *et al.* Inhibition of Plasmepsin V activity demonstrates its essential role in protein export, PfEMP1 display, and survival of malaria parasites. *PLoS Biol.* **12**, e1001897 (2014).

32 Zhu, Z. *et al.* Discovery of cyclic acylguanidines as highly potent and selective beta-site amyloid cleaving enzyme (BACE) inhibitors: Part I--inhibitor design and validation. *J. Med. Chem.* **53**, 951-965 (2010).

33 Mastrangelo, E. *et al.* Targeting the X-linked inhibitor of apoptosis protein through 4-substituted azabicyclo[5.3.0]alkane smac mimetics. Structure, activity, and recognition principles. *J. Mol. Biol.* **384**, 673-689 (2008).

34 Huber, E. M., Heinemeyer, W. & Groll, M. Bortezomib-resistant mutant proteasomes: structural and biochemical evaluation with carfilzomib and ONX 0914. *Structure* **23**, 407-417 (2015).

35 Eschenburg, S. *et al.* Crystal structure of subtilisin DY, a random mutant of subtilisin Carlsberg. *Eur. J. Biochem.* **257**, 309-318 (1998).

36 Sealy, J. M. *et al.* Design and synthesis of cell potent BACE-1 inhibitors: Structure–activity relationship of P1′ substituents. *Bioorg. Med. Chem. Lett.* **19**, 6386-6391 (2009).

37 Mitsuya, M. *et al.* Discovery of novel 3, 6-disubstituted 2-pyridinecarboxamide derivatives as GK activators. *Bioorg. Med. Chem. Lett.* **19**, 2718-2721 (2009).

38 Priestle, J. *et al.* Comparative analysis of the X-ray structures of HIV-1 and HIV-2 proteases in complex with CGP 53820, a novel pseudosymmetric inhibitor. *Structure* **3**, 381-389 (1995).

39 Cormier, A., Marchand, M., Ravelli, R. B., Knossow, M. & Gigant, B. Structural insight into the inhibition of tubulin by vinca domain peptide ligands. *EMBO Rep.* **9**, 1101-1106 (2008).

40 Papaconstantinou, M., Gandhi, P., Chen, Z., Bah, A. & Di Cera, E. Na+ binding to meizothrombin desF1. *Cell Mol. Life Sci.* **65**, 3688-3697 (2008).

41 Bernstein, N. K., Cherney, M. M., Yowell, C. A., Dame, J. B. & James, M. N. Structural insights into the activation of *P. vivax* plasmepsin. *J. Mol. Biol.* **329**, 505-524 (2003).

42 Rew, Y. *et al.* Discovery and optimization of piperidyl benzamide derivatives as a novel class of 11β-HSD1 inhibitors. *Bioorg. Med. Chem. Lett.* **19**, 1797-1801 (2009).

43 Kinoshita, T., Nakanishi, I., Sato, A. & Tada, T. True interaction mode of porcine pancreatic elastase with FR136706, a potent peptidyl inhibitor. *Bioorg. Med. Chem. Lett.* **13**, 21-24 (2003).

44 Debela, M. *et al.* Chymotryptic specificity determinants in the 1.0 Å structure of the zinc-inhibited human tissue kallikrein 7. *Proc. Natl. Acad. Sci.* **104**, 16086-16091 (2007).

45 Akif, M. *et al.* High-resolution crystal structures of Drosophila melanogaster angiotensin-converting enzyme in complex with novel inhibitors and antihypertensive drugs. *J. Mol. Biol.* **400**, 502-517 (2010).

46 Natesh, R., Schwager, S. L., Evans, H. R., Sturrock, E. D. & Acharya, K. R. Structural details on the binding of antihypertensive drugs captopril and enalaprilat to human testicular angiotensin I-converting enzyme. *Biochemistry* **43**, 8718-8724 (2004).

47 Zheng, L., Baumann, U. & Reymond, J.-L. Molecular mechanism of enantioselective proton transfer to carbon in catalytic antibody 14D9. *Proc. Natl. Acad. Sci.* **101**, 3387-3392 (2004).
